# Recovery of neuronal and network excitability after spinal cord injury and implications for spasticity

**DOI:** 10.3389/fnint.2014.00036

**Published:** 2014-05-12

**Authors:** Jessica M. D'Amico, Elizabeth G. Condliffe, Karen J. B. Martins, David J. Bennett, Monica A. Gorassini

**Affiliations:** ^1^Centre for Neuroscience, University of AlbertaEdmonton, AB, Canada; ^2^Faculty of Medicine and Dentistry, University of AlbertaEdmonton, AB, Canada; ^3^Department of Biomedical Engineering, University of AlbertaEdmonton, AB, Canada; ^4^Division of Physical Medicine and Rehabilitation, University of AlbertaEdmonton, AB, Canada; ^5^Faculty of Physical Education and Recreation, University of AlbertaEdmonton, AB, Canada; ^6^Faculty of Rehabilitation Medicine, University of AlbertaEdmonton, AB, Canada

**Keywords:** serotonin, noradrenaline, motoneuron, persistent inward currents, reflexes, spinal cord injuries

## Abstract

The state of areflexia and muscle weakness that immediately follows a spinal cord injury (SCI) is gradually replaced by the recovery of neuronal and network excitability, leading to both improvements in residual motor function and the development of spasticity. In this review we summarize recent animal and human studies that describe how motoneurons and their activation by sensory pathways become hyperexcitable to compensate for the reduction of functional activation of the spinal cord and the eventual impact on the muscle. Specifically, decreases in the inhibitory control of sensory transmission and increases in intrinsic motoneuron excitability are described. We present the idea that replacing lost patterned activation of the spinal cord by activating synaptic inputs via assisted movements, pharmacology or electrical stimulation may help to recover lost spinal inhibition. This may lead to a reduction of uncontrolled activation of the spinal cord and thus, improve its controlled activation by synaptic inputs to ultimately normalize circuit function. Increasing the excitation of the spinal cord with spared descending and/or peripheral inputs by facilitating movement, instead of suppressing it pharmacologically, may provide the best avenue to improve residual motor function and manage spasticity after SCI.

## Introduction

In the months following a spinal cord injury (SCI), 70–80% of individuals develop spasticity (Maynard et al., 1990; Skold et al., 1999), which is characterized by involuntary muscle activity such as spasms, hyperreflexia, clonus and co-contraction (Pandyan et al., 2005; Nielsen et al., 2007). There is also an increased resistance to passive stretch and the development of joint contractures, the latter characterized by reduced joint range of motion and deformity mediated, in part, by changes to tissues in the muscle and joint (Diong et al., 2012). Although spasticity can interfere with residual motor function and produce pain, it can also be useful. For example, involuntary muscle spasms (i.e., prolonged contractions) and tone in extensor muscles can facilitate walking (Skold, 2000), standing and transfers (Satkunam, 2003; Adams and Hicks, 2005). In this review, we compare evidence from both animal and human studies to discuss how muscle and non-muscle tissues respond to the different phases of SCI and how neurons and neuronal circuits increase their excitability and decrease their inhibitory capacity to compensate for the loss of descending and movement-related sensory inputs, ultimately leading to the development of spasticity. In many cases, there is good agreement with animal and human studies but also some important differences. We propose that instead of further suppressing spinal cord activity with antispastic medications, perhaps we should be doing just the opposite, by finding ways to functionally reactivate the spinal cord to restore lost inhibition and normalize neuronal and circuit function. In this way, improvements in both residual motor control and/or reductions in spasticity can occur without the unwanted side effects of antispastic medications.

## Assessments of spasticity in humans

Throughout this review, several clinical measures of spasticity are discussed as they are used to evaluate the effectiveness of different interventions (Hsieh et al., 2008). The frequently used Ashworth (Ashworth, 1964) and modified Ashworth (Bohannon and Smith, 1987) scales rate, from 0 to 4, the resistance of a relaxed, single joint to movement imposed by the evaluator throughout its full available range. The pendulum test, which also measures resistance to passive motion, records changes in knee joint angle after the relaxed leg is allowed to swing freely following its release from an extended position (Wartenberg, 1951; Bajd and Vodovnik, 1984). To measure spasticity evoked by various stimuli, the Spinal Cord Assessment Tool for Spasticity (SCATS) rates, on a scale of 0–3, the severity of low frequency (4–12 Hz) rhythmic movements of the ankle (i.e., clonus, Wallace et al., 2005) and flexor and extensor spasms in response to pinprick or rapid leg extension, respectively (Benz et al., 2005). The self-reported Penn spasm frequency scale asks patients to assess the average frequency and source of spasms from 0 (no spasms on most days) to 4 (>10 spontaneous spasms/hour) (Penn et al., 1989). However, like most of the other assessment methods, the scale is ordinal with incompletely explored reliability, validity, and responsiveness (Biering-Sorensen et al., 2006; Hsieh et al., 2008). Moreover, these assessments are limited to a single point in time during the assessment, even though spasticity varies by time of day, recent activity level, temperature and emotional status (Biering-Sorensen et al., 2006). Thus, more objective measures of spasticity, based on actual muscle activation patterns recorded over many hours/days, are needed.

## Changes in tissue properties after SCI

### Muscle

As with neurons and neuronal circuits, muscle and connective tissue also adapt to SCI. With a reduced activity and unloading after SCI in humans, muscles begin to atrophy within the first months as measured by decreased fiber diameter, cross-sectional area and volume (Scelsi et al., 1982; Lotta et al., 1991; Biering-Sorensen et al., 2009). There are also changes in the joint angle-torque relationship of some muscles that may accompany reductions in the range of joint motion. For example, in plantarflexors (but not dorsiflexors) peak twitch torque occurs at more plantarflexed joint angles in SCI participants compared to non-injured controls (McDonald et al., 2005; Pelletier and Hicks, 2010). The shift in the joint angle-torque relationship has been attributed to shortening of the muscle as a result of sarcomere loss and increases in connective tissue, but this has not been directly proven. Changes in the composition of the muscle fiber can also occur after SCI, although different results have been obtained when examining non-spastic vs. spastic muscles. Several animal models of SCI, where no clear spasticity develops, and several human studies have a reported a decrease in the proportion of the fatigue-resistant type I and less-fatigable type IIa fibers coupled with an increase in the most-fatigable type IIb or IId/x fibers [abbreviated together as IIb(x)](Scelsi et al., 1982; Lotta et al., 1991; Roy et al., 1992, 1999; Harris et al., 2007; Malisoux et al., 2007). In humans with complete SCI, the slower-to-faster fiber type transitions begin ~4–7 months post-SCI and continue until almost all fibers are type IIb(x), which can occur 2–6 years post-SCI (reviewed in Biering-Sorensen et al., 2009).

Importantly, in the studies described above, the amount of muscle activity present from day-to-day was not well described. It is reasonable to assume that muscle activity has an effect on skeletal muscle properties. As a model of this, acute transection of the sacral spinal cord in adult rats initially results in tail muscles that are inactive, but then a clear spasticity syndrome develops 2 weeks later that mimics what happens after SCI in humans (Bennett et al., 1999, 2004). Acutely, when muscles of the tail were inactive, as measured by 24-h EMG recordings, there was a transition from slow, fatigue-resistant type I to predominantly fast, fatigable type IIb(x) fibers compared to uninjured control animals (Harris et al., 2007). However, in spastic muscles with enhanced EMG activity compared to controls, the amount of atrophy measured in the myofibers was substantially reduced. Moreover, the proportion of type I to type II fibers was similar to age-matched control rats (Harris et al., 2007), although the muscles were more fatigable (Harris et al., 2006). In agreement with the rat results, participants with motor incomplete [ASIA Impairment Scale (AIS) C&D] or motor complete (AIS A&B) injuries having marked spasticity exhibited stimulus-torque responses characteristic of plantarflexor muscles with *slow* contractile properties (Hidler et al., 2002; Pelletier and Hicks, 2010). Additionally, in participants with motor complete and incomplete SCI (AIS B&C), muscle cross-sectional area was positively correlated to modified Ashworth scores (Gorgey and Dudley, 2008). In contrast, muscles of individuals with low measures of spasticity displayed faster contractile properties compared to control muscles, in line with the human studies described earlier (reviewed in Biering-Sorensen et al., 2009).

Collectively, these results indicate that similar to exercise (Roy et al., 1999), the involuntary activity present in spastic muscles allows some retention of normal muscle fiber type composition and contractile properties; however, the muscles still remain more fatigable. Increased fatigability after SCI in humans appears to be related to changes in the muscle (Klein et al., 2006). For example, the metabolic capacity of muscle, as measured by oxidative enzyme activity (Shields, 1995; Wang et al., 1999) and concentrations of Na^+^/K^+^-ATPase (Ditor et al., 2004), is correlated to the amount of fatigue resistance in individuals with SCI (Shields, 1995). In summary, although spastic muscle activity promotes slow contractile and fiber-type properties of muscle, it is not enough to preserve fatigue resistance. Treatments such as intensive exercise (Roy et al., 1999) or electrical stimulation (Rochester et al., 1995; Gerrits et al., 2002, 2003) are likely also needed to improve muscle endurance via increases in oxidative capacities of the muscle (Gerrits et al., 2003).

### Muscle connective tissue

Similar to muscle, remodeling of non-muscle tissue also occurs after SCI. For example, after SCI in humans atrophic myofibers become replaced by adipocytes, collagen, and other amorphous substances (Scelsi et al., 1982; Olsson et al., 2006). It is thought that these morphological changes increase the intrinsic stiffness of the muscle (Mirbagheri et al., 2001; Schleip et al., 2006). However, in individuals with increased passive tension in the vastus lateralis at the whole muscle and muscle fiber level, specifically in type IIb(x) fibers, there were *no* changes in passive tension at the myofibril level (Olsson et al., 2006; Malisoux et al., 2007). Likewise, the muscle protein titin, a main contributor to passive tension (Horowits et al., 1986; Labeit and Kolmerer, 1995), remained unchanged in spastic muscles when compared to control muscles, as did the properties of the intermediate filaments (Olsson et al., 2006). Thus, as muscle atrophy occurs, the increased passive tension evident at the whole muscle and muscle fiber level is likely not due to structural changes of the myofibril, but rather due, in part, to the replacement of myofibrils by amorphous substances such as collagen and connective tissue (Scelsi et al., 1982; Olsson et al., 2006). Moreover, adaptations to extracellular and joint tissues may also contribute to joint stiffness after SCI, but these changes have only been demonstrated in immobilization models (Gracies, 2005).

In summary, changes in muscle and non-muscle tissue, combined with adaptations to preserved spinal circuitry as described next, likely contributes to spastic motor behaviors after SCI. For example, increases in muscle stiffness and changes in the joint angles that produce optimal torque may contribute to the decreased threshold and increased gain of the stretch reflex pathway that mediate, in part, the oscillatory activation of muscles during clonus (de Vlugt et al., 2012).

## Changes in motoneuron properties after SCI

### Spinal shock

Immediately after injury, the spinal cord enters a state of “spinal shock” (Ditunno et al., 2004; Dietz, 2010) that is characterized by severe muscle paralysis, flaccid muscle tone (Bastian, 1890; Sherrington, 1899, 1909) and an initial loss of reflexes and sensation caudal to the lesion (Leis et al., 1996; Little et al., 1999; Ditunno et al., 2004; Dietz, 2010). The duration and severity of spinal shock differs between species, lasting only minutes to hours in the frog (Hall, 1850), cat (Sherrington, 1899; Hunt et al., 1963; Chambers et al., 1966) and dog (Sherrington, 1899; Fulton and Sherrington, 1932) but up to 2 weeks in the monkey (Sherrington, 1899; Fulton and Sherrington, 1932; Hunt et al., 1963; McCouch et al., 1966), rat (Bennett et al., 1999) and several weeks in humans (Ko et al., 1999). In humans, all reflexes are absent only for the first 24 h after SCI (Phase 1) (Ditunno et al., 2004), which coincides with the first 2 weeks of areflexia in the rat tail model of SCI (Bennett et al., 1999). By 1–3 days post-injury, human H-reflexes and cutaneous reflexes begin to return (Diamantopoulos and Olsen, 1967; Ashby et al., 1974; Ko et al., 1999; Hiersemenzel et al., 2000) while a delayed plantar response also develops (Phase 2: Ko et al., 1999; Ditunno et al., 2004). Phase 3, lasting 4 days to 1 month, is characterized by a gradual increase in all reflexes with the disappearance of the abnormal plantar response. Spinal shock in humans is considered resolved by 1–6 months post-injury with eventual development of hyperreflexia, clonus and muscle spasms (Ko et al., 1999; Ditunno et al., 2004). Similarly in the rat, hypertonus in flexor and extensor muscles, clonus and hyperreflexia (cutaneous tail flick) develops by 2 months post-injury (Bennett et al., 1999).

Several factors contribute to the initial suppression of spinal cord excitability after SCI. For example, there is a slight hyperpolarization (2–6 mV) of the resting membrane potential in cat (Cope et al., 1980; Schadt and Barnes, 1980) and rat motoneurons (Li et al., 2007), which may explain why antidromic activation of human motoneurons, as measured by F-waves, is difficult acutely after SCI (Ashby et al., 1974). In contrast, H-reflexes, but not tendon tap reflexes, recover during spinal shock, as shown in both the cat (Hunt et al., 1963; Zapata, 1966) and human (Weaver et al., 1963; Hiersemenzel et al., 2000). This suggests that fusimotor drive is also reduced acutely after SCI given that tendon taps rely on muscle spindle excitability whereas H-reflexes do not. Pre-synaptic inhibition of primary afferents is also increased in spinal shock, as measured by increases in primary afferent depolarization in cats (Quevedo et al., 1993).

**Figure 1:**
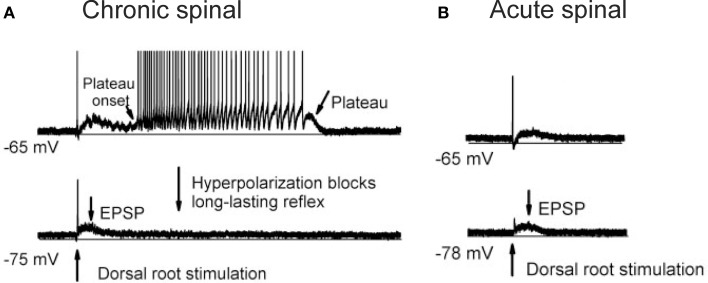
**Re-emergence of PICs after chronic SCI. (A)** Re-emergence of PICs in a completely transected (S2) rat after chronic (>50 days post-injury) SCI. Motoneuron recording in response to single pulse (3 × 's sensory threshold) dorsal root stimulation. At resting membrane potential (−65 mV, top trace), PIC activation produces a plateau potential and self-sustained firing. Hyperpolarization of the motoneuron (−75 mV, bottom trace) deactivates the PIC, eliminating the long-lasting reflex (LLR or spasm) and reveals the long (1 s) EPSP. **(B)** Spinal shock in acutely-spinalized rat (hours after injury), same preparation and experimental set-up as in **(A)**. Note absence of long-lasting reflex response at resting membrane potential (−65 mV, top trace) due to elimination of PICs acutely after injury. The long-duration motoneuron EPSP (~1 s) appears acutely after injury as revealed in hyperpolarized motoneuron (−78 mV, bottom trace). Taken from Li et al. (2004a).

One of the more prominent contributors to spinal shock is likely the disappearance of dendritic, voltage-activated sodium and calcium persistent inward currents (PICs) in motoneurons acutely after SCI. PICs contain a TTX-sensitive persistent Na current and a low-voltage activated, slowly inactivating L-type Ca current (CaV1.3) that is sensitive to nimodipine (Li and Bennett, 2003). PICs act to amplify synaptic inputs (Lee and Heckman, 2000) and because they are activated sub-threshold to cell firing (Bennett et al., 1998), PICs aid in the secure and rapid recruitment of motoneurons (Lee and Heckman, 2000). After recruitment, PICs produce a sustained depolarization, or plateau potential, which can last for many seconds to produce self-sustained firing of the motoneuron in the presence of reduced synaptic inputs (Hounsgaard et al., 1984, 1986; Crill and Schwindt, 1986; Carlin et al., 2000; Powers and Binder, 2003; reviewed in Heckman et al., 2005). For example, a 1-s long, sub-threshold depolarization of the motoneuron by a sensory-evoked, excitatory post-synaptic potential (EPSP) activates a plateau potential (Figure 1A), which then keeps the motoneuron discharging for many seconds even though the synaptic input (EPSP, see hyperpolarized trace) has subsided. During spinal shock, PICs are no longer activated in response to sensory stimulation. In motoneurons of acutely injured rats (Figure 1B), the same 1-s long EPSP does not activate a plateau potential and subsequently, self-sustained firing does not occur. Thus, the acute disappearance of PICs dramatically reduces the excitability of the motoneuron as the amplification and prolongation of synaptic inputs does not occur. This, coupled with motoneuron hyperpolarization, increases in pre-synaptic inhibition and decreases in background synaptic and gamma motoneuron drive, renders the motoneuron and spinal circuits unexcitable in the first days and weeks after SCI.

### Recovery of motoneuron PICs

In the weeks and months that follow a SCI, neurons and neuronal circuits below the injury adapt to inactivity by increasing intrinsic excitability. This recovered excitability is marked in human SCI by increases in residual muscle strength, H-reflexes, F-wave persistence and the return of flexor reflex responses (Leis et al., 1996; Little et al., 1999; Hiersemenzel et al., 2000). In addition to changes in the transmission of sensory inputs (as discussed later on), motoneuron PICs also re-emerge in the weeks following a SCI to contribute to both the recovery of motor function and the development of spasticity. For example, the motoneuron described previously in Figure 1A actually comes from a rat whose sacral spinal cord was completely severed 2 months previously and whose tail muscles developed prolonged muscle spasms. Thus, the self-sustained activity in this motoneuron, which is mediated by the PIC and subsequent plateau potential, reflects the involuntary muscle spasms that develop in these animals. The long time course of the plateau potential, which can last for several seconds, is mainly produced by the slowly inactivating CaPIC given that the NaPIC inactivates over a few seconds (Lee and Heckman, 2001; Miles et al., 2005; Harvey et al., 2006) and blocking CaPICs greatly reduces long-lasting reflexes (LLR), i.e., spasms (Murray et al., 2011b).

**Figure 2:**
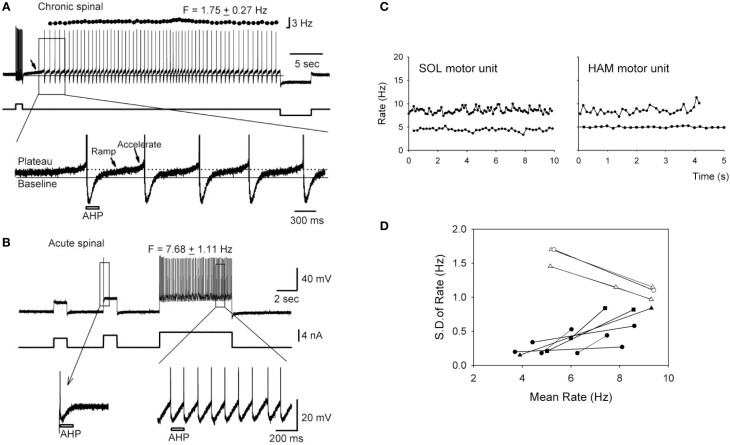
**Slow, steady firing in rat and human motoneurons after chronic SCI. (A)** *Top trace:* Firing response of a motoneuron from a chronic spinal rat in response to a brief depolarizing current pulse (~2 nA). Note low mean rate (1.75 Hz) and small standard deviation (±0.27 Hz) in firing. *Bottom trace:* Magnification of top trace indicated by rectangle. Plateau activation due to NaPIC is indicated by dotted line, and baseline membrane potential (Vm) is represented by solid black line. Note the ramp and acceleration (arrows) in Vm after each AHP due to NaPIC, producing interspike intervals much longer than the duration of the AHP (gray bar). **(B)** *Top trace:* Firing response of motoneuron from an acute spinal rat without plateau potentials in response to large (~4–5 nA) depolarizing current injections. Note increased firing rate (7.68 Hz) during large current pulse. *Bottom trace:* Magnification of top trace as indicated by rectangle. Interspike intervals are now closer to the duration of the AHP (gray bar) and lack NaPIC-mediated ramp in Vm. **(A**,**B)** taken from Li et al. (2004a). **(C)** Slow, steady firing rate of spontaneously-active soleus (SOL) and hamstrings (HAM) motor units from two participants with incomplete SCI (5 Hz firing). Note increase in firing rate and variability during superimposed voluntary activation of the muscles (10 Hz firing). **(D)** Relationship between the firing rate variability [standard deviation (SD)] and mean rate of motor units from SCI participants (black symbols) and control, uninjured participants (white symbols). **(C,D)** taken from Gorassini et al. (2004).

Instead of producing prolonged plateau potentials, the persistent NaPIC facilitates repetitive discharge of the motoneuron and mediates the slow, but steady firing that is characteristic of spontaneous motor unit discharge in individuals with chronic SCI (e.g., 5 Hz discharge in Figure 2C; Gorassini et al., 2004; see also Zijdewind and Thomas, 2012). For example, in motoneurons from chronically spinalized rats, the NaPIC is activated shortly after the upswing on the afterhyperpolarization (AHP) to produce a slow ramp and then acceleration in membrane potential (Vm) which then triggers an action potential (see bottom trace in Figure 2A). Following this, during the fast depolarizing phase of the action potential (spike), the NaPIC deactivates but then re-activates on the upsweep of the next AHP to slowly ramp up the Vm and trigger another spike. In this way, the NaPIC helps to regeneratively fire the motoneuron at interspike intervals that are much longer than the duration of the AHP (marked by bar), especially under conditions of weak or absent depolarizing synaptic drive (Li and Bennett, 2007). Thus, repetitive activation of the NaPIC produces the slow but steady motoneuron firing often observed after SCI (e.g., 1.8 Hz firing in Figure 2A). In motoneurons from acutely injured rats, there is little to no NaPIC as demonstrated by the absence of a slow acceleration in Vm after the AHP (first inset in Figure 2B, bottom trace). Because of this, repetitive firing only occurs during a strong depolarizing drive, as occurs during a large current injection (see third current pulse in Figure 2B). The large current injection provides sufficient acceleration of the Vm after the AHP to bring the motoneuron to threshold (second inset, bottom trace). Thus, without the contribution of the NaPIC, the motoneuron can only fire under a strong depolarizing drive, with interspike intervals that are close to the duration of the AHP, resulting in faster firing rates (e.g., 7.7 Hz firing in Figure 2B).

Evidence for the activation of NaPICs in mediating the slow, but steady firing in human motoneurons after SCI is demonstrated by the increase in firing rate variability when volitional drive is superimposed on a spontaneously active motor unit (Figure 2C). Here, the steady but slow discharge of the motor unit at 5 Hz is thought to be mediated by the regenerative activation of a strong NaPIC under conditions of low, involuntary depolarizing synaptic drive, similar to rat motoneurons. With the introduction of volitional drive, synaptic noise to the motoneuron increases and subsequently, both the firing rate of the motor unit (e.g., 10 Hz in Figure 2C), along with its spike-to-spike variability, increase (black symbols in Figure 2D). This suggests that the faster, more variable firing is produced by the noisy depolarization from the voluntary synaptic drive that recruits the motoneuron at random times during the slow, NaPIC-mediated ramp in Vm. The relationship between firing rate and firing variability is opposite in motor units from uninjured control participants during increasing levels of voluntary drive (white symbols in Figure 2D). Under low levels of voluntary drive, firing rates are low (~5 Hz) but variable due to low levels of synaptic noise (EPSPs) that sporadically brings the Vm to threshold after the AHP (Matthews, 1996). However, when voluntary drive increases, so does the mean depolarizing drive to the motoneuron and this helps to more consistently accelerate the Vm to threshold after the AHP (as in Figure 2B). Thus, during strong voluntary drive in uninjured individuals, firing rate variability at high rates decreases because the interspike interval (ISI) is more dependent upon the stable AHP rather than the synaptic noise (Miles et al., 2005). For example, firing rate variability is low and plateaus beyond 10 Hz (ISI's ≤ 100 ms, Person and Kudina, 1971), likely because the ~100 ms AHP occupies a larger proportion of the ISI compared to at lower rates (ISI's > 100 ms) where random synaptic noise has more opportunity to recruit the motoneuron during the NaPIC-mediated, slow ramp in Vm. Interestingly, when the firing rate in the SCI and uninjured motor units both reach ~10 Hz, the firing rate variability in the two groups become similar, suggesting that at rates >10 Hz, the AHP is the main determinant of the ISI.

In summary, after SCI both CaPIC-mediated plateau potentials and NaPIC-mediated regenerative firing allow motoneurons to exhibit prolonged, involuntary firing in response to brief or low-levels of depolarizing synaptic drive.

### Estimating PICs from human motor unit recordings

**Figure 3:**
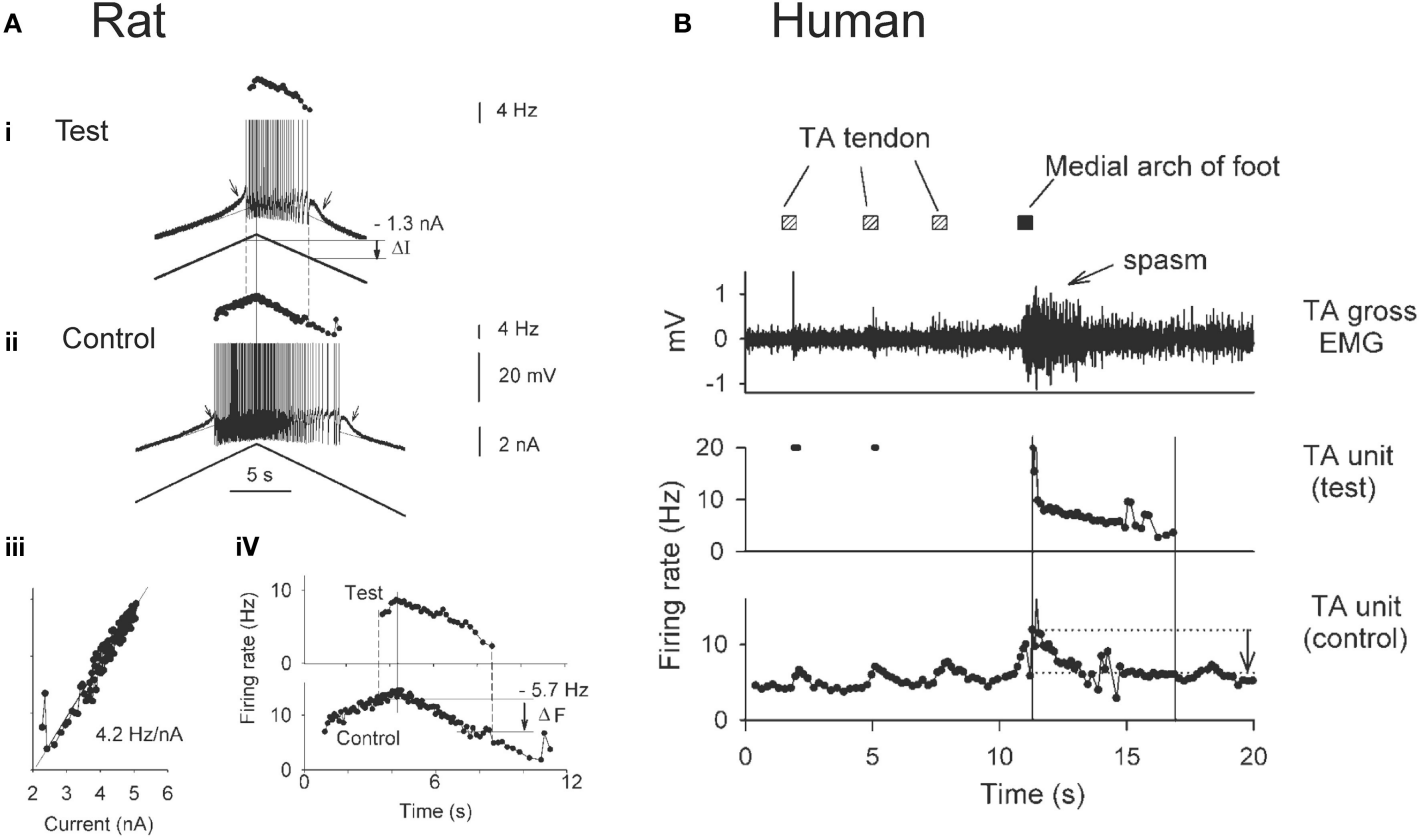
**Estimating PICs in rat and human motoneurons. (A)** Rat motoneurons: Paired motor unit analysis (ΔF) technique in rat motoneurons. **(i,ii)** Firing rate profiles of a control and test motoneuron in response to the same triangular current injection. Measurement of PIC as the difference in current input at recruitment and derecruitment of the test motoneuron (dashed lines): ΔI value. **(iii)** Linear relationship between the firing rate and injected current in the control motoneuron. **(iv)** The control motoneuron is serving as a measure of input to the test motoneuron. Measurement of the PIC as the difference in firing rate of the control motoneuron at recruitment and derecruitment (dashed lines) of the test motoneuron: ΔF value. **(B)** Human motoneurons: ΔF during muscle spasm in human SCI. *Top trace*: Tibialis anterior (TA) surface EMG during sustained dorsiflexion and application of transient vibration to TA tendon (gray squares) and medial arch of the foot (black square). *Middle trace*: Firing rate of a higher-threshold TA test unit in response to the vibrations. *Bottom trace*: Firing rate profile of tonically active lower-threshold control motor unit. ΔF is measured as the difference in control unit rate at derecruitment and recruitment of test motor unit (vertical lines and arrow). **(A,B)** taken from Gorassini et al. (2004).

The use of paired motor unit recordings in humans has provided indirect evidence that PICs contribute to the involuntary activation of motoneurons during muscle spasms in chronic SCI. The basis of estimating PIC activation by using the firing profiles of two motor units (motoneurons) is demonstrated from direct intracellular recordings in the rat (Figure 3A). For example, the amplitude of the PIC can be estimated indirectly during current clamp when the motoneuron is activated by a triangular-shaped current injection (Bennett et al., 2001). Once the PICs are activated, the added depolarization provided by the PIC allows the motoneuron to fire at levels of injected current (input) below that needed to recruit the motoneuron (below top horizontal line: Figure 3Ai), with cell firing stopping only when the injected current is substantially reduced (at second horizontal line). The difference in injected current between derecruitment and recruitment of the motoneuron (ΔI) is then used as a measure of the PIC amplitude because it is the amount of current that needs to be removed to counteract the added depolarization provided by the PIC and stop the motoneuron from firing.

Using the firing rate profiles of two motoneurons to estimate the amplitude of the PIC requires that both cells receive a common input and that the firing rate of one of the motoneurons (e.g., control motoneuron in Figure 3Aii) serves as an estimate of the input to the second motoneuron (e.g., test motoneuron). This is possible because the firing rate of a motoneuron is linearly related to the amount of current injection (or synaptic input) it receives once the PIC is fully activated, as shown in Figure 3Aiii. Thus, when using the firing rate (F) of the control motoneuron as a measure of input to the test motoneuron (Figure 3Aiv), rather than the injected current, the difference in firing rate of the control motoneuron at derecruitment and recruitment of the test motoneuron (dashed lines) corresponds to the reduction in input that is required to counteract the added depolarization from the PIC (Fderecruitment-Frecruitment = ΔF). Dividing the ΔF value (5.7 Hz) by the slope of the F-I relationship measured in Figure 3Aiii (4.2 Hz/nA) results in an estimation of current (1.4 nA) that is close to the amplitude of the PIC measured from the injected current profile (1.3 nA in Figure 3Ai).

By using the firing rate profiles of two motoneurons (motor units) as described above, the contribution of PICs to the activation of motoneurons during involuntary muscles spasms has been estimated in participants with SCI (Figure 3B). Here the firing rate of a lower-threshold control motor unit from the tibialis anterior (TA) muscle that is activated by vibration to the TA tendon (Figure 3B, bottom trace) is used as a measure of input to the TA motoneuron pool and to a higher threshold test motor unit. As evidence of this, vibration of the TA tendon (hatched squares) produced a transient increase in the firing rate of the control unit and a transient recruitment of a higher-threshold test unit (Figure 3B: middle trace). Vibration of the tendon did not produce a strong enough afferent input to evoke a muscle spasm. However, vibration of the medial arch of the foot (Figure 3B: black square) produced a greater increase in the firing rate of the tonically-active control unit, signifying greater synaptic input to the motor units, and consequently resulted in stable recruitment of the higher-threshold test unit. In fact, the newly recruited test unit continued to fire well after the removal of the vibratory input, and exhibited sustained firing even at levels of synaptic input (as measured by the firing rate of the control unit) well below the level needed to recruit the test unit (difference between horizontal lines, arrow indicates ΔF). This difference indicates the activation of a PIC. By comparing the estimated PIC amplitude (ΔF = 5 Hz) to the total amount of rate modulation in the test unit during a moderate contraction or involuntary spasm (~12 Hz), it is estimated that the PIC provides ~40% of the depolarizing drive to the motoneuron during self-sustained firing (Gorassini et al., 2004) and therefore, plays a major role in driving involuntary muscle spasms after SCI in humans. During stronger muscle spasms, peak firing rates of motor units can reach as high as 35 Hz (Thomas and Ross, 1997) where the relative contribution of PIC and synaptic activation of the motoneuron is unknown.

Using the paired motor unit technique to estimate PIC amplitude (ΔF) in human participants relies on the important assumptions that: (1) the firing rate of the control unit is an accurate representation of its synaptic input and (2) the control unit receives the same synaptic drive as the test unit (Gorassini et al., 2004). Thus, it is important to measure the firing rate of the control unit during periods of linear input-output which likely occurs during moderate rates of discharge (5–20 Hz) and durations of spike trains (10–20 s), to avoid firing rate saturation and adaptation, respectively (Sawczuk et al., 1997; Brownstone, 2006). These values are based on firing rate properties of sacral rat motoneurons that are similar to firing properties of human motor units in the lower limb (Li et al., 2004a). Likewise, firing rates of the control unit during the first and last 1–2 s of activity should be avoided as initial activation of PICs and AHP conductances, respectively, could affect the firing rate of the motoneuron (Bennett et al., 1998; Wienecke et al., 2009). Finally, the correlation between the smoothed firing rate of a control and test motor unit should be high (correlation coefficient > 0.8) to ensure that both units are receiving a common synaptic drive (De Luca and Erim, 2002). Thus, ΔF measures can provide a reasonable estimate of PIC amplitude when the above conditions are followed. However, one must keep in mind that it is not possible to directly measure the linearity of the input-output relation of human motoneurons.

### Modulation of motoneuron PICs by monoamine receptors

In addition to being voltage-sensitive, PICs require the concomitant activation of serotonergic (5-HT) or noradrenergic (NA) receptors located on the motoneurons. Serotonin is synthesized from the amino acid, L-tryptophan, via the enzymes L-tryptophan hydroxylase and amino acid decarboxylase (AADC) (Feldman et al., 1997). Neuronal excitability is modified by 5-HT via seven main types of receptors, 5-HT_1−7_, which are all G-protein coupled receptors (GPCRs) except for the ligand-activated 5-HT_3_ receptor (Boess and Martin, 1994; Hoyer et al., 2002; Nichols and Nichols, 2008). PICs on motoneurons are modulated by 5-HT_2B/C_ receptors specifically (Miller et al., 1996; Murray et al., 2011a). Noradrenaline, like dopamine and adrenaline, is a catecholamine synthesized by adrenal chromaffin cells and sympathetic nerves and is converted from the amino acid L-tyrosine into L-Dopa via tyrosine hydroxylase (Kopin, 1968). L-Dopa is then converted into dopamine via AADC and subsequently into noradrenaline via the enzyme dopamine beta hydroxylase (DβH). Noradrenaline exerts its actions via three main different types of GPCRs: α1, α2, and β subtypes (Ahlquist, 1980; Bylund, 1992), where α1 receptors are coupled to downstream Gq pathways and affect motoneuron PICs, similar to 5-HT_2_ receptors (Gershengorn, 1989; Minneman and Esbenshade, 1994; Lee and Heckman, 1996) and α2 receptors are coupled to downstream Gi pathways to affect sensory transmission, similar to 5-HT_1_ receptors (Ruffolo et al., 1991; Murray et al., 2011b). The major source of serotonin to the spinal cord, as identified in rodents, comes from 5-HT containing neurons in the medullary raphe pallidus (B1), raphe obscuris (B2), raphe magnus (B3) and from adjacent parts of the reticular formation (reviewed in Schmidt and Jordan, 2000). The dorsal horn is mainly innervated by neurons from the raphe magnus, whereas motoneurons in the ventral horn receive 5-HT inputs mainly from the raphe pallidus and obscuris. The A5, A6 (locus coeruleus) and A7 NA cell groups in the pontine region provide the major source of NA fibers to the spinal cord, with a predominant innervation of the dorsal horn by A6 axons and of the ventral horn by A7 axons (Bruinstroop et al., 2012).

**Figure 4:**
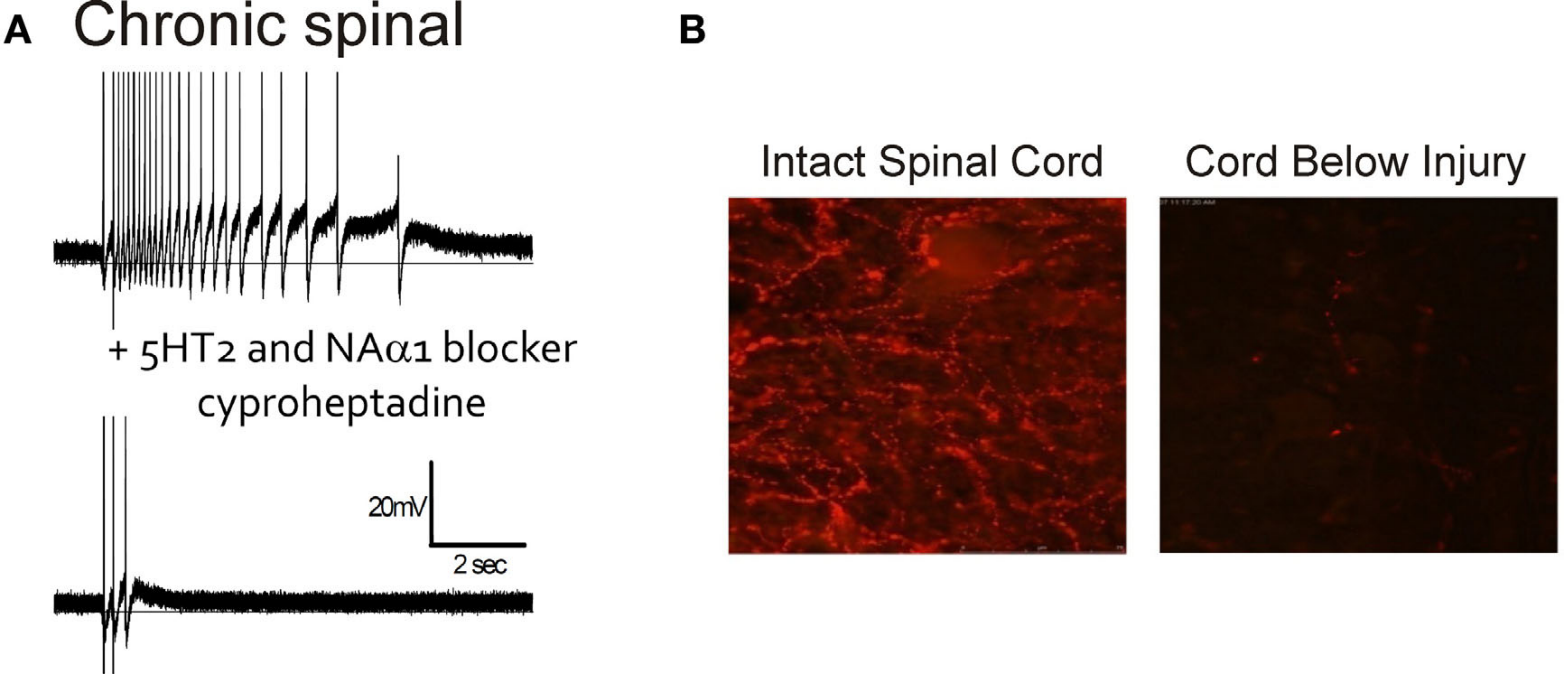
**Endogenous activation of 5HT2/NAα1 receptors with little monoamines below injury. (A)** *Top trace*: Intracellular recording from motoneuron of chronic spinal rat. Single-pulse, dorsal root stimulation evokes long-lasting plateau potential and self-sustained firing. *Bottom trace*: Blocking of 5-HT_2_ and NAα_1_ receptors with cyproheptadine eliminates PIC-mediated plateau potential and self-sustained firing, leaving only sensory-mediated activation of motoneuron. **(B)** Immunofluorescence imaging of residual serotonergic fibers (beaded red) labeled with Texas-Red in spinal cords above (left) and below (right) a complete spinal transection from a rat spinalized 2 months previously. **(B)** was taken from Murray et al. (2010).

The role of 5-HT and NA receptors in facilitating motoneuron PICs was first demonstrated in the decerebrate cat where PICs and sustained reflex responses were blocked by methysergide; a 5-HT and NA receptor blocker (Hounsgaard et al., 1986, 1988). Likewise, immediately following a complete spinal transection where descending sources of 5-HT and NA were abolished (i.e., acute injury), PICs were reduced or eliminated but could be subsequently restored with an intravenous application of 5-HTP, a 5-HT precursor (Hounsgaard et al., 1988) or by L-DOPA (Conway et al., 1988), indicating loss of 5-HT_2_ and NAα_1_ receptor activity. In contrast to acute SCI, serotonergic and NA receptors are tonically activated on motoneurons below a complete, chronic transection, as evidenced by the reduction of PIC activation and spasms by cyproheptadine, a 5-HT_2_ and NAα_1_ receptor blocker (Figure 4A). Interestingly, the activation of these monoaminergic receptors occurs even though there is a marked disappearance of both 5-HT and NA fibers and their monoamines below the lesion within 7 and 9 days after injury (Carlsson et al., 1963; Anden et al., 1964). For example, in rat spinal cords there is a reduction in immunolabeling for 5-HT below the lesion 2 months after a complete transection (Figure 4B). As will be described below, the reactivation of 5-HT_2_ and NAα1 receptors on the motoneuron, and the recovery of PICs in chronic SCI, results from an increase in monoamine receptors that can be activated without the binding of 5-HT and NA. This “self-activation” of 5-HT_2_ and NAα_1_ receptors is one strategy the spinal cord uses to regain its lost excitability.

### Constitutively-active 5-HT_2_ and NAα_1_ receptors

**Figure 5:**
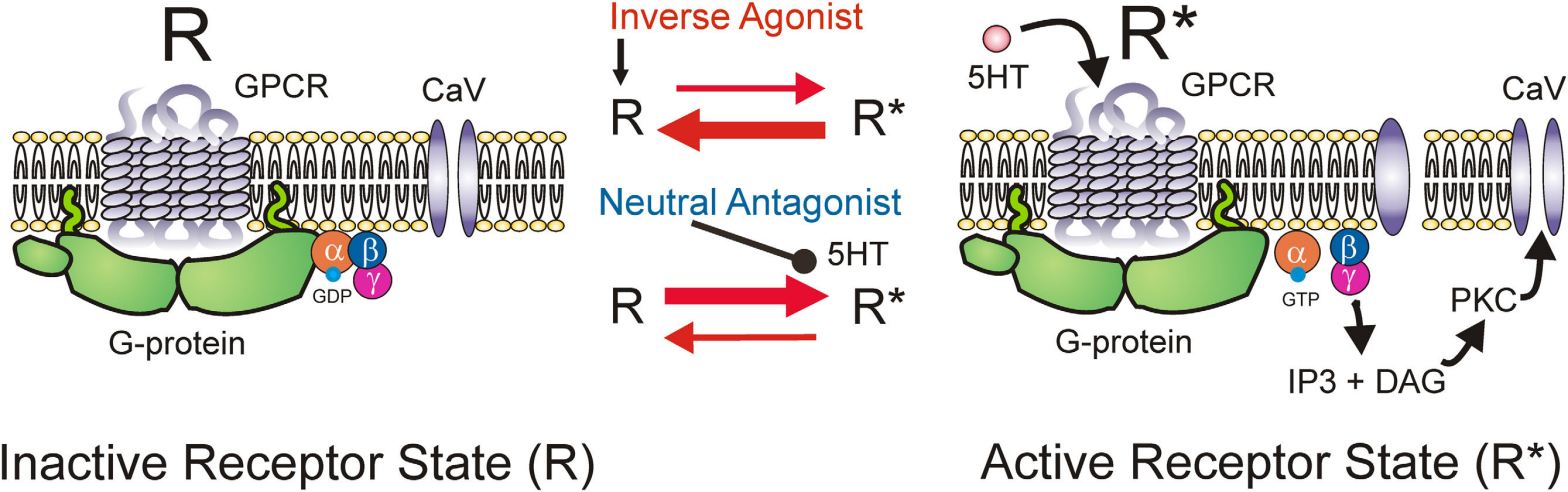
**Mechanism of inverse agonists and neutral antagonists**. Receptor state equilibrium: Inactive state (R, left panel) of G-protein coupled receptor (GPCR) where α, β, and γ subunits of the G-protein and downstream pathways are not activated. Active receptor state (R^*^, right panel) where the α-subunit is released by facilitating exchange of GDP for GTP. Dissociated α-subunit activates phospholipase C (PLC, not shown) resulting in the hydrolysis of phosphatidylinositol bisphosphate into the secondary messengers inositol trisphosphate (IP_3_) and diacylglycerol (DAG). IP_3_ increases calcium mobilization via release of intracellular, IP_3_-regulated calcium stores and DAG activates the downstream protein kinase C (PKC) which subsequently phosphorylates and activates voltage-gated channels such as the Na and Ca channels mediating the PICs.

5-HT_2_ and NAα_1_ receptors are coupled to a hetero-trimeric G protein which consists of an α, β, and γ subunit (Raymond et al., 2001; Heckman et al., 2003). Activation of GPCRs induces a conformational change in the receptor complex which results in the release of the α-subunit by facilitating the exchange of GDP for GTP. The dissociated α-subunit then acts to stimulate the β isoform of the enzyme phospholipase C (PLC), resulting in the hydrolysis of phosphatidylinositol bisphosphate into the secondary messengers inositol trisphosphate (IP_3_) and diacylglycerol (DAG). IP_3_ increases calcium mobilization via release of intracellular, IP_3_-regulated calcium stores and DAG activates the downstream protein kinase C (PKC) (Mizuno and Itoh, 2009) which subsequently phosphorylates and activates voltage-gated channels such as the Na and Ca channels mediating the PICs. An interesting characteristic of GPCRs, like the 5-HT_2_ and NAα_1_ receptors, is their ability to display constitutive receptor activity (Gether et al., 1997; Seifert and Wenzel-Seifert, 2002; Berg et al., 2005; Navailles et al., 2006). GPCRs exist in equilibrium between inactive (R) and active (R^*^) receptor states (Figure 5). In the active state, the receptor is coupled to its G-protein and can activate downstream signaling pathways, such as those which facilitate the motoneuron PIC. Typically receptors enter their active state only when the appropriate ligand binds to the receptor complex (ligand-activation). However, different isoforms of a receptor can also spontaneously enter their active state without activation by a ligand and this is termed *constitutive receptor activity*.

**Figure 6:**
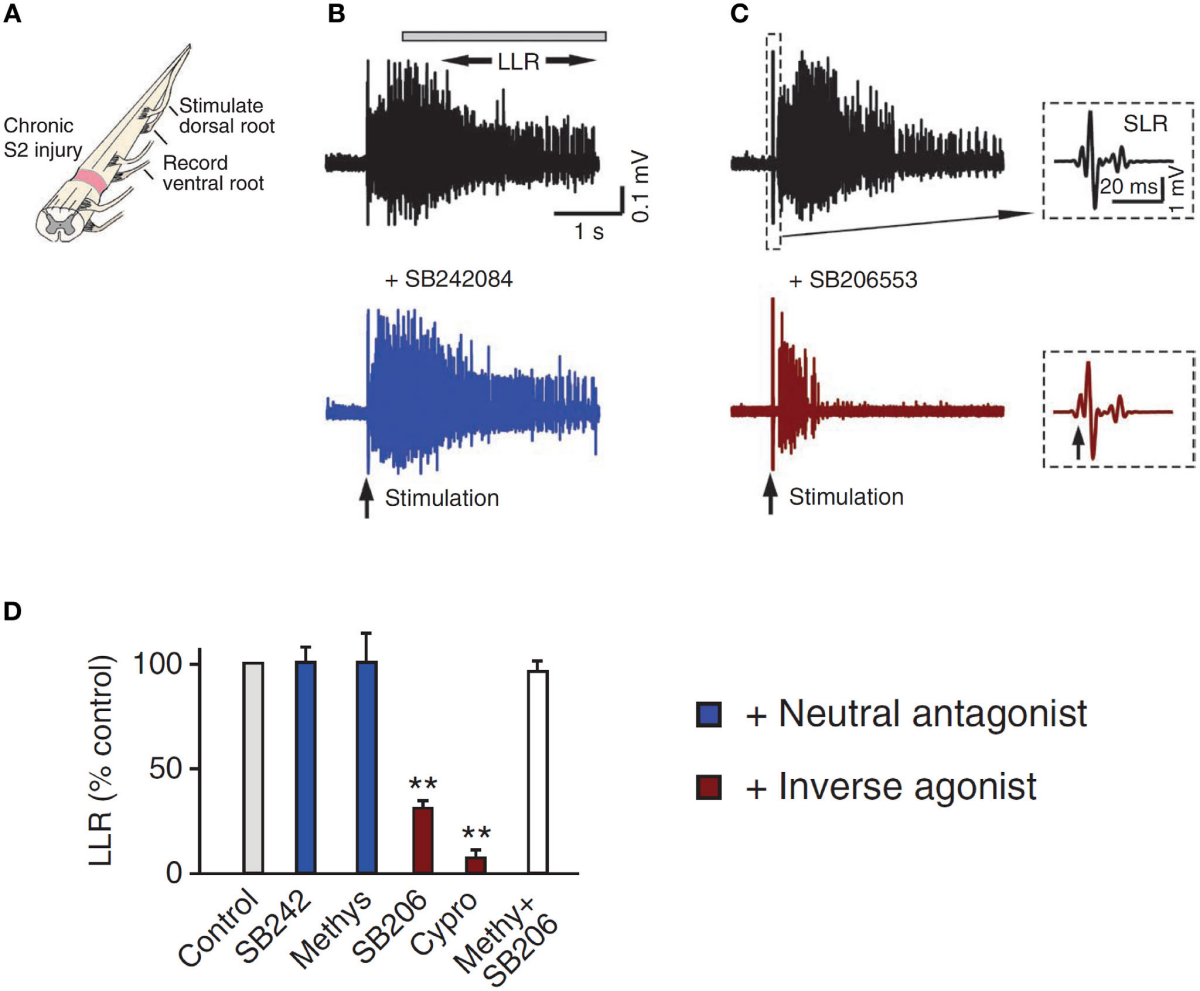
**Constitutive receptor activity in chronic SCI rat. (A)** *In vitro* ventral root recordings in response to single-pulse (3 × T) dorsal root stimulation in chronically spinalized (S2) rats. **(B)** PIC-mediated long-lasting reflex (LLR: 500–4000 ms after stimulation) before (black trace) and after (blue trace) application of the neutral antagonist SB242084. **(C)** Evoked LLR before (black trace) and after (red trace) application of the inverse agonist SB206553. Inset shows the sensory evoked, short-latency reflex (SLR: 10–40 ms) that is not mediated by the slow activating PICs or affected by the inverse agonist. **(D)** Group means of the LLR (expressed as a % of pre-drug values) after application of the neutral antagonists (blue), inverse agonists (red), and application of inverse agonist after the receptor is first blocked with the neutral antagonist (white) to render the inverse agonist ineffective. Taken from Murray et al. (2010). ^**^p < 0.01.

5-HT_2C_ and NAα_1_ receptors demonstrate constitutive receptor activity, as measured by basal IP_3_ levels and through pharmacological methods in human (HEK -293) and monkey (COS-7) kidney cells and in cultured mouse cortical neurons (Herrick-Davis et al., 2000; Rauser et al., 2001; Berg et al., 2005; Navailles et al., 2006; Chanrion et al., 2008). To examine if constitutive activity in 5-HT_2_ and NAα_1_ receptors mediates the recovery of motoneuron PICs in chronic injury, the effects of two types of antagonists, an inverse agonist and a neutral antagonist, on PIC-mediated LLR (spasms) has been examined, first in completely transected rats (Murray et al., 2010) and then in humans with SCI (D'Amico et al., 2013b). An *inverse agonist* stabilizes the receptor in its inactive resting state (R), preventing it from entering into its active state (R^*^). It also blocks other agonists from binding to the receptor and thus, inverse agonists block both constitutive receptor activity *and* conventional ligand-activation of the receptor (Figure 5; Westphal and Sanders-Bush, 1994; Herrick-Davis et al., 2000; Chanrion et al., 2008). In contrast, a *neutral antagonist* works by only blocking the ligand activation of the receptor. The 5-HT_2_ and NAα1 receptor inverse agonist, cyproheptadine, and the selective 5-HT_2_ receptor inverse agonist SB206553, both reduced PIC-mediated, LLR in chronically-spinalized rats (Figures 6C,D: red traces/bars) and had no effect on sensory-mediated, short-lasting reflexes (SLR). In contrast, the neutral antagonists methysergide and SB242084, which only block the ligand from activating the 5-HT_2_ and NAα_1_ receptors, had no effect on PIC-mediated responses (Figures 6B,D: blue traces/bars). Because the inverse agonist reduced 5-HT_2_ and NAα_1_ receptor activity and its downstream effects (e.g., reduction of PIC-mediated LLR) and the neutral antagonist had no effect, then the effects of the inverse agonists can be attributed solely to blocking receptors that were constitutively active. Furthermore, mRNA analysis revealed an upregulation in the INI isoform of the 5-HT_2C_ receptor which displays the highest degree of constitutive receptor activity (Murray et al., 2010). Consistent with the mRNA findings, 5HT_2C_ immunoreactivity is increased by 60% in motoneurons below a complete transection (Ren et al., 2013). Evidence for constitutively active NAα_1_ receptors was also found whereby the inverse agonists prazosin and WB4101 significantly reduced PIC-mediated responses in chronic spinal rats whereas the neutral antagonists methysergide (5-HT_2_/NAα_1_) and REC (NAα_1_) had no effect (Rank et al., 2011). Thus, following complete SCI in the chronic animal, the recovery of PIC activation in the motoneuron occurs as a result of the emergence of constitutive 5-HT2 and NAα1 receptor activity.

**Figure 7:**
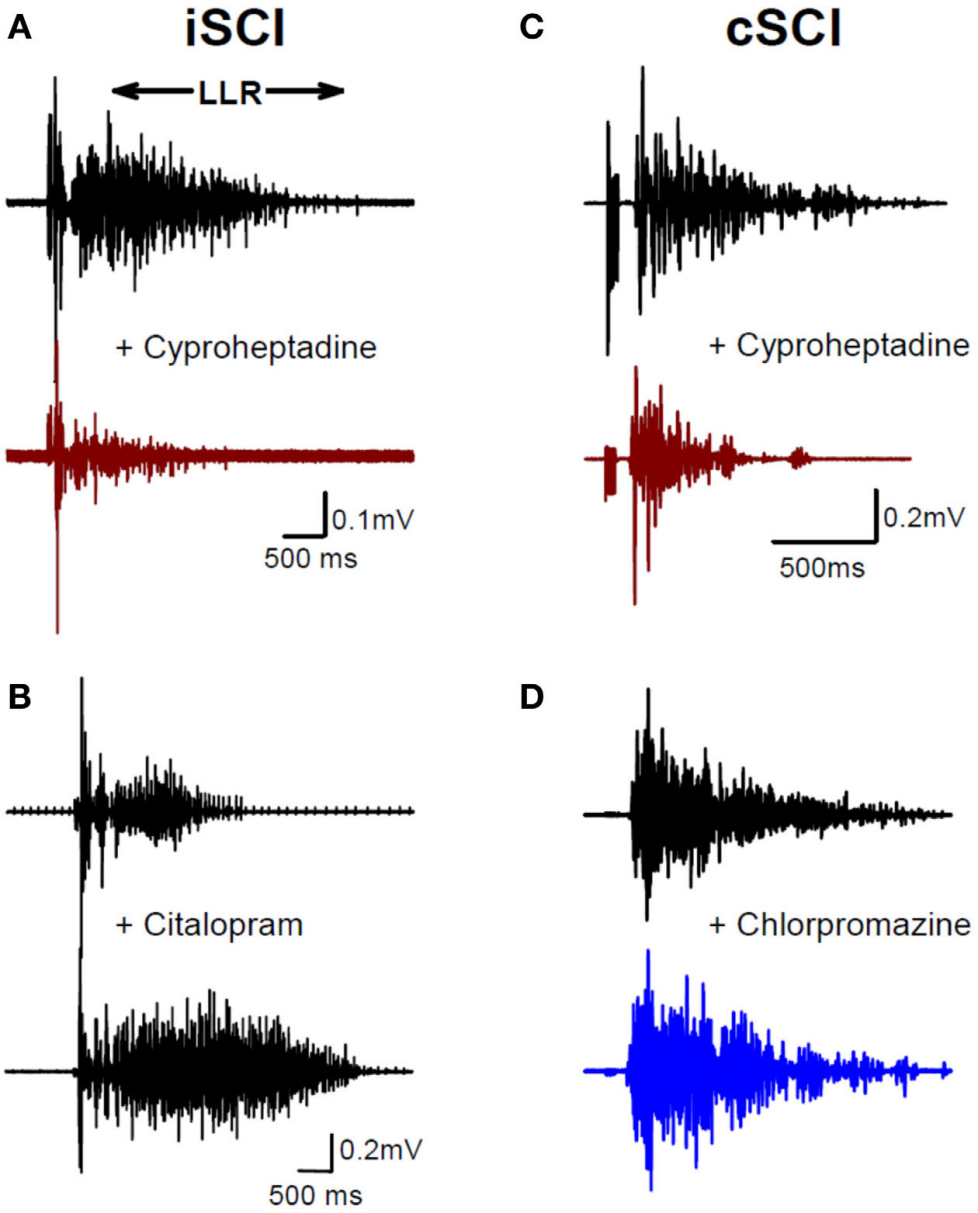
**Constitutive receptor activity in chronic human SCI. (A)** Inverse agonist cyproheptadine in a motor incomplete SCI participant (iSCI, AIS C). Unrectified surface EMG during long-lasting reflex (LLR) evoked in the TA muscle from medial arch stimulation before (top trace) and after (bottom trace) 8 mg of oral cyproheptadine. Note reduction in the presumably PIC-mediated long-lasting reflex (LLR: 500 ms after stimulation onwards). **(B)** Serotonin re-uptake inhibitor citalopram in iSCI (AIS C) participant. Long-lasting reflex (LLR) recorded in TA muscle before (top trace) and after (bottom trace) 20 mg of oral citalopram. **(C)** Same as in **(A)** but for 12 mg oral dose of cyproheptadine in motor and sensory complete SCI participant (cSCI, AIS A). **(D)** Effect of neutral antagonist chlorpromazine (12.5 mg) on PIC-mediated LLR in same cSCI participant. **(A–C)** taken from D'Amico et al. (2013b).

To examine the emergence of constitutively-active monoaminergic receptors after SCI in humans, similar pharmacological methods have been employed in individuals with both motor incomplete (iSCI: AIS C&D) and motor complete (cSCI: AIS A&B) injury (D'Amico et al., 2013b). Oral administration of cyproheptadine, the inverse agonist to 5-HT_2_ and NAα_1_ receptors, also reduced PIC-mediated LLR in participants with *incomplete* SCI (Figure 7A) and had no effect on the sensory-mediated SLRs similar to the rat (D'Amico et al., 2013b). In these same participants oral intake of citalopram, a selective serotonin reuptake inhibitor, increased the LLR (Figure 7B), indicating that residual levels of serotonin were present below the injury and likely facilitated motoneuron PICs via ligand activation of the 5-HT_2_ receptors (see also Thompson and Hornby, 2013). Similar to participants with incomplete SCI, participants with motor *complete* injuries also displayed tonic activation of 5-HT_2_ and NAα_1_ receptors given that cyproheptadine also reduced PIC-mediated LLR (Figure 7C). However, oral intake of the neutral antagonist, chlorpromazine, had no effect on LLR (Figure 7D), even though it was effective in reducing PIC-mediated ΔF measures in uninjured controls having intact 5-HT and NA fibers (D'Amico et al., 2013b). Based on these two findings, participants with *motor complete* SCI appeared not to have residual levels of 5-HT and NA below the lesion so that PICs were solely facilitated by constitutive activity in 5-HT_2_ and NAα_1_ receptors, similar to the complete SCI model in rats. How the facilitation or control of these monoamine receptors may be used to promote motor recovery or control spasticity, respectively, will be discussed at the end of this review.

## Changes in sensory transmission to motoneurons after SCI

### Emergence of long-duration EPSPs

In addition to adaptive changes in the motoneuron, the transmission of sensory inputs to the motoneuron is also affected following SCI. One of the main changes is the loss or reduction of early, inhibitory post-synaptic potentials (IPSPs) and the conversion to pure, prolonged EPSPs in response to brief, sensory stimulation (Baker and Chandler, 1987). As demonstrated in Figure 1, following acute or chronic SCI, a single pulse of electrical stimulation to a sacral dorsal root, which contains predominantly cutaneous afferents, produced a very long (~1 s) EPSP that was dependent upon the activation of NMDA channels (Bennett et al., 2001). The profile of this EPSP was revealed by blocking voltage-dependent conductances with cell hyperpolarization so that the synaptic activation profile of the motoneuron predominated (Li et al., 2004a). This 1-s EPSP provided a long enough depolarization of the motoneuron to activate the CaPIC, the latter requiring at least 500 ms of synaptic depolarization to activate (Moritz et al., 2007; Murray et al., 2011b). Thus, when the motoneuron PIC recovers after chronic SCI, the long EPSP can trigger plateau potentials and self-sustained firing that is unchecked due to the loss of descending and intrinsic inhibition of the motoneuron, resulting in the activation of involuntary muscle spasms.

**Figure 8:**
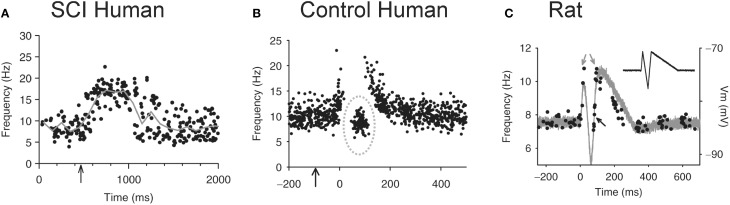
**Peristimulus frequencygrams (PSF) in SCI and uninjured participants and in rat. (A)** PSF of TA motor units recorded in incomplete SCI participant (AIS C) in response to stimulation of the medial arch of the foot (time marked by arrow, 0.2 ms pulse width, 3 pulses, 300 Hz, ~50 mA) while participant maintained tonic dorsiflexion. Mean rate indicated by solid gray line. **(B)** PSF recorded from uninjured control participant in response to same medial arch stimulation. Note shorter duration of response, pause and cluster of motor unit action potentials as marked by the dashed gray circle. **(C)** PSF (black dots) recorded in rat motoneuron that mimics the PSF obtained in the uninjured control participant from **(B)**. The intracellular current injection profile required to obtain this PSF is shown in inset and the resulting Vm response (gray trace) to this injected profile in a hyperpolarized motoneuron. Taken from Norton et al. (2008).

The loss of IPSPs and conversion to pure EPSPs in response to cutaneomuscular stimulation is also evident in the activation profile of motoneurons (motor units) recorded in participants with chronic, incomplete SCI (Norton et al., 2008) using the peristimulus frequencygram (PSF) technique (Awiszus et al., 1991; Turker and Powers, 1999, 2003). To construct a PSF, the instantaneous firing rate of a tonically activated motor unit is plotted time-locked to a sensory stimulus (Figure 8). Because the firing rate of a motoneuron mainly reflects the net current reaching the soma (Baldissera et al., 1982; Powers et al., 1992), any modulation in firing rate from a sensory stimulus should reflect the underlying shape of the EPSP or IPSP (Turker and Powers, 1999, 2003). For example, the firing rate of a tonically active motor unit recorded from the TA muscle of an incomplete SCI participant increased above baseline for a duration of ~1 s in response to stimulating cutaneomuscular afferents from the medial arch of the foot. This was most likely due to an EPSP lasting for ~1 s, similar to that measured in motoneurons from chronically spinalized rats (Figure 1A). In a control participant (Figure 8B), the PSF only lasted for ~300 ms, with a pause in firing after the initial acceleration in rate and an interposed cluster of action potentials at firing rates near or below the mean background rate (gray circle: Figure 8B). The shape of this PSF was likely mediated by a 300 ms long EPSP that contained a brief, interposed IPSP. To demonstrate this, a current profile was injected into a hyperpolarized motoneuron from a chronic spinal rat (inset in Figure 8C) to produce the hypothesized PSP (gray trace in Figure 8C). When a steady current was injected into the motoneuron to produce tonic firing and the simulated current profile was then added, the resulting PSF (black dots in Figure 8C) was similar to the PSF recorded in the control participant (Figure 8B). Thus, it has been proposed that, unlike uninjured control participants, robust IPSPs from cutaneomuscular afferent stimulation are not activated in motoneurons of SCI participants and are replaced by prolonged EPSPs. These 1 s-long EPSPs produce a long enough depolarization of the motoneuron to activate CaPICs and trigger self-sustained firing, resulting in the activation of unchecked, involuntary muscle spasms.

The animal and human studies described above mainly examined changes in the transmission of cutaneous reflex pathways. As described next, transmission in other sensory pathways is also altered after SCI. Generally, transmission is increased in excitatory reflex pathways and reduced in inhibitory reflex pathways.

### Pre-synaptic inhibition

**Figure 9:**
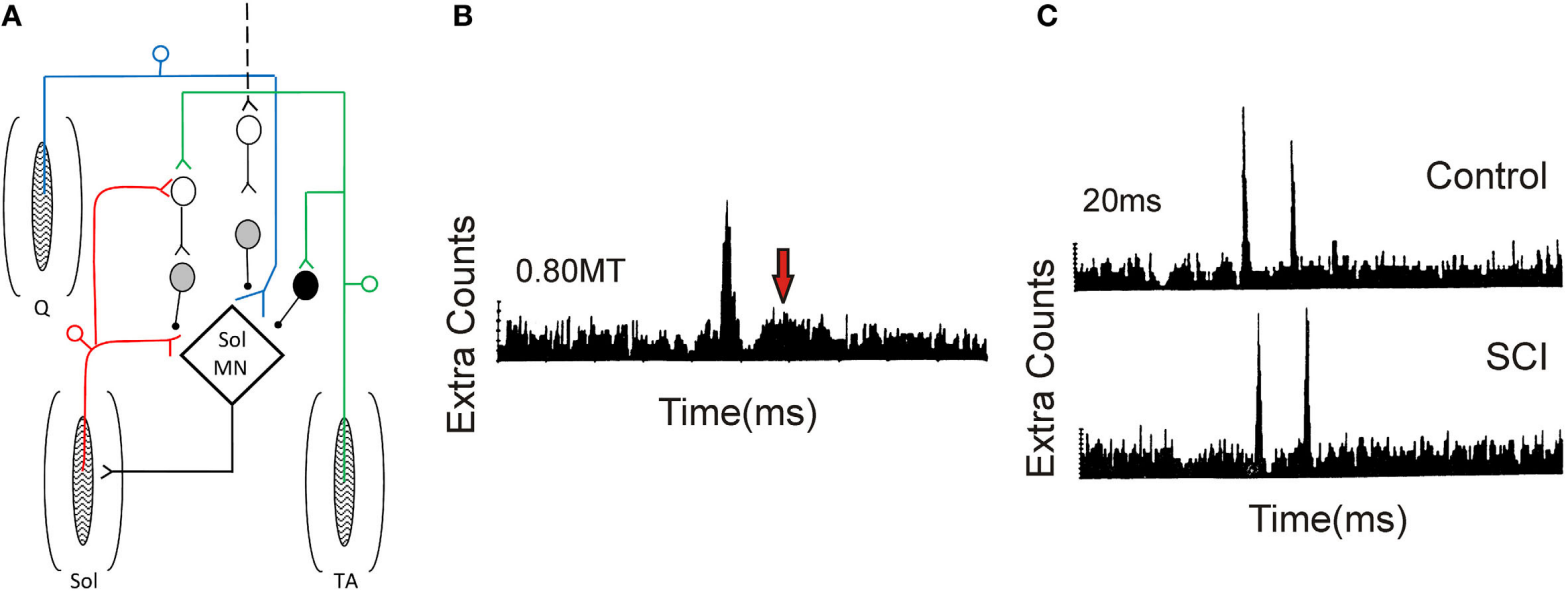
**Pre-synaptic and reciprocal inhibition in humans. (A)** Pre-synaptic and reciprocal inhibitory pathways. *Red trace*: Monosynaptic Ia pathway to soleus (Sol, lower pathway) and homonymous activation of PAD interneurons (upper pathway). PAD interneurons marked by gray circles. *Blue trace*: Heteronymous, monosynaptic facilitation of soleus motoneurons by quadriceps (Q) Ia afferents that are tonically inhibited by PAD Ins, the latter activated by descending pathways (dashed line). *Green trace*: Reciprocal inhibitory pathway from tibialis anterior (TA) afferents onto soleus Ia reciprocal inhibitory interneuron (black circle) and onto soleus PAD interneuron via second order interneuron (D1/D2 inhibition). **(B)** PSTH obtained from a SCI participant in response to low-threshold electrical stimulation of the posterior tibial nerve (bottom red pathway in **A**). Note second peak in PSTH (arrow). **(C)** Decrease of second PSTH peak in response to paired activation (20 ms ISI) of posterior tibial nerve in uninjured control (top trace) but not SCI (bottom trace) participant indicative of reduced pre-synaptic inhibition (top red pathway in **A**). **(B,C)** were modified from Mailis and Ashby (1990).

Pre-synaptic inhibition effectively modulates the efficacy of afferent transmission to the motoneuron via primary afferent depolarizing interneurons (PAD INs, gray neurons in Figure 9A). Although pre-synaptic inhibition can occur at all afferent terminals, studies in humans have mainly focused on pre-synaptic inhibition of Ia afferents. The mechanisms of pre-synaptic inhibition involve the activation of fast, ionotropic γ-aminobutyric acid_A_ (GABA_A_) receptors and slow, metabotropic GABA_B_ receptors. Activation of GABA_A_ receptors on primary afferent terminals produces an efflux of Cl^−^ resulting in primary afferent depolarization and subsequently, reducing the size of the propagating action potential. This ultimately results in less calcium influx and less neurotransmitter release (Rudomin and Schmidt, 1999; Seki et al., 2003; Rudomin, 2009). In contrast, G-protein coupled GABA_B_ receptors directly modulate the calcium channels in afferent terminals, resulting in less calcium influx and subsequently, less neurotransmitter release (Rudomin and Schmidt, 1999; Castro et al., 2006; Soto et al., 2006).

Mailis and Ashby (1990) examined transmission from Ia afferents to motoneurons after SCI by recording motor unit action potentials in the soleus muscle in response to low-intensity (~0.8 xMT) posterior tibial nerve stimulation (lower red path in Figure 9). In only the most spastic SCI participants, the peak amplitude of the post-stimulus time histogram (PSTH) at the Ia monosynaptic latency was augmented (first peak in Figure 9B). In addition, there was the emergence of a second peak 11–15 ms later (at arrow in Figure 9B), suggesting the emergence of an oligosynaptic, group I excitatory pathway. To examine if this enhanced response was due to a reduction in pre-synaptic inhibition on the Ia afferent terminals, paired electrical stimulation of homonymous afferents from the soleus muscle was used (Birnbaum and Ashby, 1982; Lev-Tov et al., 1983). A prior conditioning stimulus (0.8 xMT) was applied to the posterior tibial nerve to activate PAD INs (upper red pathway in Figure 9), which in turn pre-synaptically inhibited Ia afferents with monosynaptic connections onto soleus motoneurons (lower red pathway). Here, the first peak of the PSTH was produced by the arrival of Ia inputs from the first conditioning volley and the second peak from the test volley (Figure 9C). In controls, the second peak in the PSTH was reduced in comparison to the first peak at interstimulus intervals (ISIs) of 20 and 50 ms, likely due to pre-synaptic inhibition of Ia afferents from the first conditioning volley (upper red pathway). Reduction of the second peak was smaller or absent in the SCI participants (Figure 9C), indicating a reduction in homonymous, pre-synaptic inhibition (Mailis and Ashby, 1990).

Reduction of pre-synaptic inhibition from *heteronymous* nerves, using the techniques of Hultborn et al. (1987) and Meunier and Pierrot-Deseilligny (1989), was also examined after SCI. Excitatory monosynaptic inputs from quadriceps Ia afferents (blue pathway in Figure 9A) can facilitate the soleus H-reflex (lower red pathway) but the amount of this facilitation is modulated by the amount of pre-synaptic inhibition that is tonically present on the quadriceps Ia afferent terminals, likely from descending pathways (marked as black dashed line, Figure 9A). Heteronymous facilitation of the soleus H-reflex by quadriceps Ia afferents (4–5 xMT, ~80 ms ISI) was greater in SCI participants compared to uninjured controls, indicating reduced levels of background pre-synaptic inhibition after SCI (Faist et al., 1994). Pre-synaptic inhibition of soleus Ia afferent terminals through PAD interneurons can also be activated by afferents supplying the antagonist tibialis anterior muscle (TA, Figure 9A, green pathway). In uninjured controls, conditioning the TA afferents produces two phases of inhibition onto soleus motoneurons: an early D1 inhibition at ISIs of 10–25 ms and a late, longer-lasting D2 inhibition at ISIs of 70–200 ms (Mizuno et al., 1971). A pre-synaptic site for the inhibition was proposed since the same conditioning volleys did not suppress cortical motor evoked potentials (MEPs) in the soleus, thereby excluding post-synaptic inhibitory mechanisms (Capaday et al., 1995; Faist et al., 1996). When an H-reflex in the flexor carpi radialis (FCR) muscle was conditioned by a prior stimulation to the antagonist radial nerve (0.95 xMT, 13 ms ISI), there was no early D1 inhibition in the agonist H-reflex in tetraplegic participants, indicating reduced pre-synaptic inhibition of Ia afferents in the median nerve (Aymard et al., 2000). In summary, pre-synaptic inhibition appears to be significantly reduced after SCI, likely resulting, in part, from decreased descending facilitation of PAD INs (Figure 9A). However, there is no correlation between the reduction in pre-synaptic inhibition and Ashworth scores of spasticity (Faist et al., 1994).

### Reciprocal inhibition

Reciprocal Ia inhibition is a disynaptic pathway through Ia interneurons (black neuron, Figure 9A) that is activated by Ia afferents from the agonist (e.g., TA) muscle and inhibits motoneurons of the antagonist (e.g., soleus) muscle. Activation of Ia inhibitory interneurons ensures that antagonist muscles remain relaxed during contraction of the agonist muscle, preventing undesired co-contractions. Reciprocal inhibition can be measured by conditioning a test H-reflex evoked in the antagonist muscle with a low-threshold stimulation (0.8–1.0 xMT, ISIs 2–4 ms) to the nerve supplying the agonist muscle (Kots and Zhukov, 1971; Mizuno et al., 1971; Crone et al., 1987). In SCI participants, suppression of the soleus H-reflex by a prior conditioning stimulation of the common peroneal nerve supplying the TA muscle was greatly reduced compared to control participants and, in some cases, replaced by reciprocal facilitation (Crone et al., 2003). Although it is apparent that reduced reciprocal inhibition may contribute to increased motoneuron excitability after SCI, there is no correlation between the strength of reciprocal inhibition and the degree of spasticity, as measured by the Ashworth scale (Crone et al., 1994; Nielsen et al., 2007).

### Post-activation depression

Post-activation (or rate-dependant) depression (PAD_2_) of H-reflexes occurs when the same (homonymous) Ia afferent pathway is repetitively activated (Crone and Nielsen, 1989). PAD_2_ produces a strong, long-lasting (~10 s) inhibition of H-reflexes and motoneuron activity (Magladery et al., 1952; Rothwell et al., 1986; Crone and Nielsen, 1989; Hultborn et al., 1996). The mechanism of PAD_2_ is unknown and may include decreases in neurotransmitter release at the Ia-motoneuron synapse (Hultborn et al., 1996; Kohn et al., 1997) or frequency-dependent facilitation of inhibitory spinal interneurons (Boulenguez et al., 2010). The level of PAD_2_ can be measured by examining the gradual decrease in H-reflex size when trains of reflexes are elicited at incrementing frequencies between 1 and 10 Hz (Lloyd and Wilson, 1957). PAD_2_ is reduced in individuals with SCI (Schindler-Ivens and Shields, 2000) and can be increased by a single bout of treadmill training (Trimble et al., 1998). Additionally, PAD_2_ can be measured by examining the reduction of H-reflexes by homonymous tendon vibration because the vibration repeatedly activates the same Ia afferents recruited during the H-reflex. Tendon vibration failed to attenuate H-reflexes in participants with chronic SCI compared to uninjured controls, again indicating reduced PAD_2_ (and/or classical pre-synaptic inhibition) as a possible contributor to enhanced sensory transmission and the triggering of muscle spasms after SCI (Calancie et al., 1993).

### Flexor withdrawal reflexes

Flexor reflex afferent (FRA) pathways include group II, joint and cutaneous afferents that activate ipsilateral flexors and contralateral extensors and inhibit ipsilateral extensors. In uninjured participants, noxious cutaneous stimulation produces an excitatory, short-latency (~50–70 ms) flexor withdrawal response with a duration of ~50 ms (Pedersen, 1954; Shahani and Young, 1971). In participants with motor complete SCI, this short latency response is not present and is replaced by a long-latency (>100 ms) excitation of flexors at both high and low intensities of stimulation (Roby-Brami and Bussel, 1987). A long-latency FRA response can also be obtained in uninjured participants but only at high stimulation intensities (Shahani and Young, 1971). In spinalized cats the early and late FRA responses are produced from distinct pathways whereby the early FRA pathway tonically inhibits the late FRA pathway. This inhibition can be released by the application of L-DOPA and NA, resulting in the emergence of long FRA responses that are similar to those present in individuals with complete SCI (Anden et al., 1964, 1966a,b; Jankowska et al., 1967; Lundberg, 1979). The development of constitutive NAα1 receptor activity on interneurons in the FRA pathway, which has been shown to occur on motoneurons (Rank et al., 2011), may contribute to the unmasking of long FRA reflexes after motor complete SCI in humans.

## Mechanisms for reduced spinal inhibition after SCI

The excitability of several inhibitory spinal pathways is reduced after SCI and likely contributes to the prolonged EPSPs that readily trigger PICs and spasms. As discussed below, inhibition and the regulation of excitatory sensory pathways is influenced by several factors.

### Depolarization of chloride reversal potential

**Figure 10:**
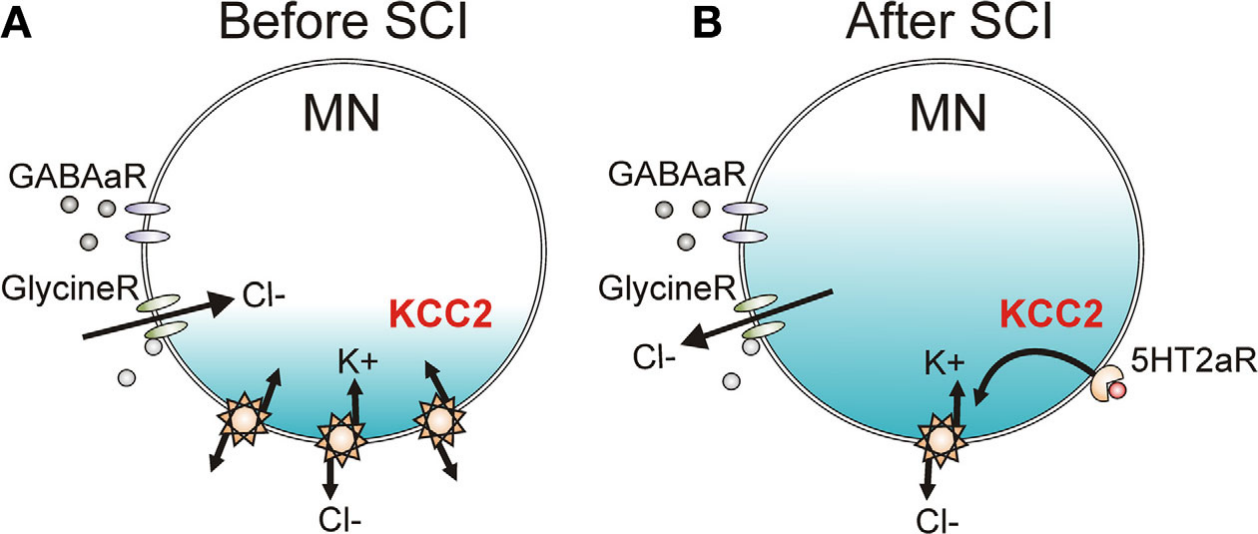
**KCC2 cotransporter and chloride equilibrium before and after SCI. (A)** A potassium chloride cotransporter (KCC2) removes chloride (Cl^−^) from the motoneuron (MN) in exchange for potassium (K^+^) to maintain Cl^−^ equilibrium potential below resting membrane potential, allowing Cl^−^ influx and MN hyperpolarization during activation of GABA and Glycine receptors (R). **(B)** Downregulation of KCC2 expression in motoneurons after SCI increases intracellular Cl^−^ concentration, depolarizing Cl^−^ equilibrium potential to above rest. This produces efflux of Cl^−^ and depolarization of MN during activation of GABA and Glycine receptors. Activation of 5-HT_2A_ receptors increases cell membrane expression of KCC2 after SCI to restore endogenous inhibition.

The strength of IPSP activation in neurons is modulated by the activity of a potassium-chloride cotransporter (KCC2, Figure 10A) which removes chloride from the neuron in exchange for potassium to maintain the chloride equilibrium potential below rest at ~75 mV (Price et al., 2005). When GABA_A_ and glycine receptors are activated, Cl^−^ flows into the neuron to produce IPSPs due to the low intracellular concentration of Cl^−^. Following SCI, there is a downregulation and decreased membrane insertion of KCC2 (Boulenguez et al., 2010), resulting in an increase in intracellular Cl^−^ concentration and an ~10 mV depolarization of the Cl^−^ reversal potential in 6–8 day old rats (Figure 10B). In adult rats, the depolarization of the Cl^−^ reversal potential is less at ~5 mV (Murray et al., 2011b). The 10 mV depolarization of the Cl^−^ reversal potential to above the resting membrane potential in neonates subsequently results in a switch from GABA_A_ and glycine-mediated inhibition to excitation because chloride moves out of the cell rather than into the cell during activation of the receptors (Boulenguez et al., 2010). Such a reversal was not seen in adult rats but the 5 mV depolarization of the Cl^−^ reversal potential to near rest likely shifts the balance of EPSP activation over IPSP activation after SCI, as shown for the PSF profiles in the human (Figure 8; Norton et al., 2008). Functionally, the reduction of KCC2 activity results in a decrease of rate-dependent depression (or PAD_2_) of the H-reflex in rats (Boulenguez et al., 2010) and may mediate the reduction of other spinal inhibitory networks such as those activated during reciprocal inhibition and cutaneomuscular reflexes. Interestingly, rate-dependent depression (or PAD_2_) of the H-reflex can be restored via activation of TrkB receptors with BDNF after SCI (but not in intact animals), which also produces an upregulation of the KCC2 cotransporter (Boulenguez et al., 2010). Activation of 5-HT_2A_ receptors on the motoneuron, via a calcium-independent PKC pathway, also restores KCC2 expression and rate-dependent depression of the H-reflex after SCI in rodents (Bos et al., 2013). Thus, strategies which enhance KCC2 activity may restore intrinsic inhibition to the spinal cord to reduce spasticity after SCI. The specific 5-HT_2A_ receptor agonist used in this study (TCB-2) is not yet available for human use and it is likely that other 5-HT_2A_ receptor agonists (e.g., DOI) would have hallucinogenic effects (Sadzot et al., 1989).

### Disinhibition from decreased monoamine receptor activation

**Figure 11:**
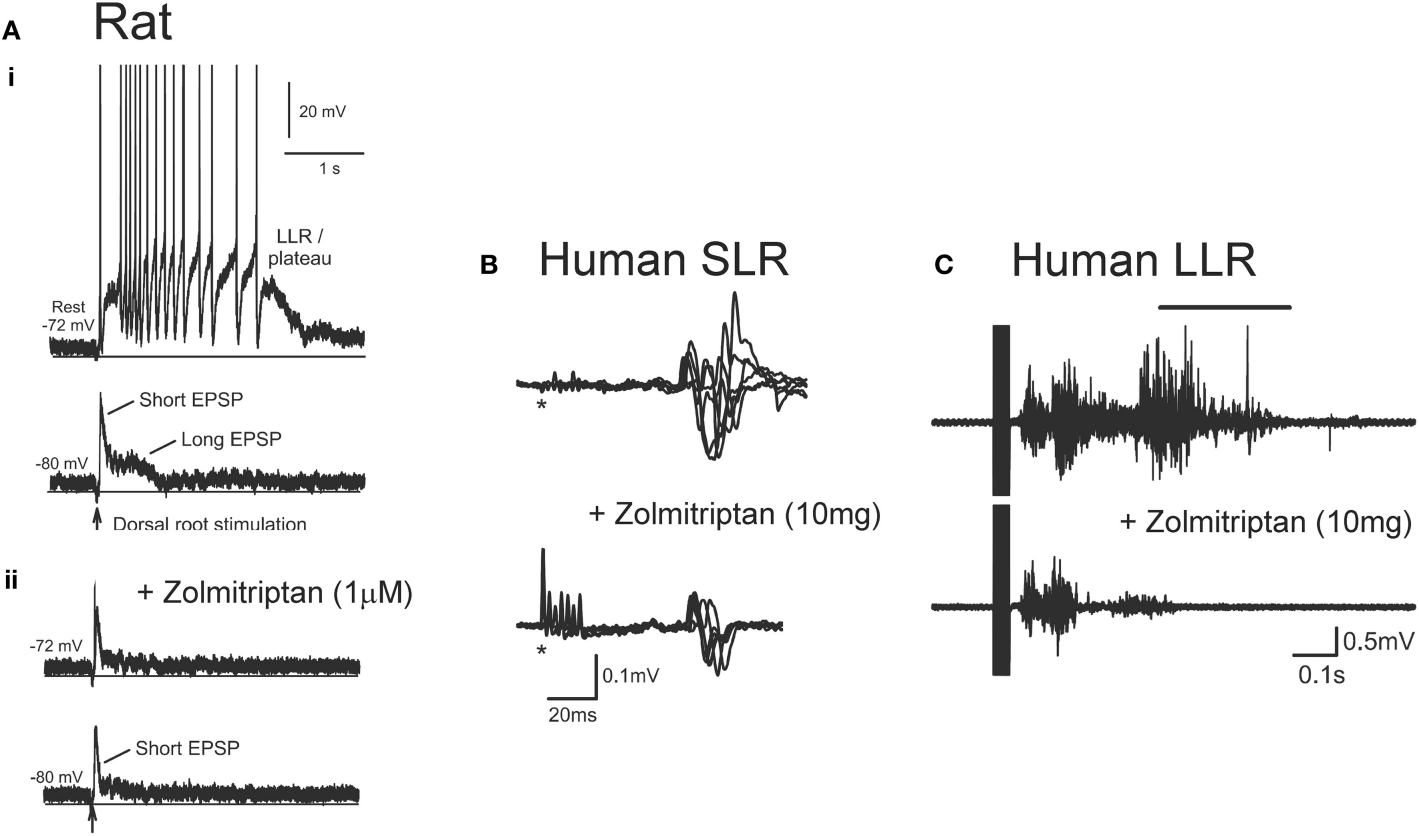
**Effect of zolmitriptan on sensory transmission in SCI rat and human. (A)** Effects of zolmitriptan in chronically injured rat motoneurons. **(i)***Top trace*: Intracellular recording from chronic spinal rat motoneuron at rest (−72 mV) where single-pulse dorsal root stimulation produces PIC-mediated long-lasting reflex (LLR). *Bottom trace*: same motoneuron hyperpolarized to −80 mV to reveal short and long EPSP in response to dorsal root stimulation. **(ii)** Recording from same motoneuron in **(i)** at rest (top trace) and during hyperpolarization (bottom trace) after application of the 5HT_1B/D_ receptor agonist zolmitriptan. Note elimination of the long-duration EPSP which prevents the triggering of the PIC-mediated long-lasting reflex. Taken from Murray et al. (2011b). **(B)** Unrectified EMG showing short- lasting reflex (SLR: first 50 ms of reflex response) recorded from the TA muscle in a single SCI participantbefore (top trace) and after (bottom trace) 10 mg oral zolmitriptan in response to medial arch stimulation. ^*^Marks time of stimulation. **(C)** Unrectified TA EMG before (top trace) and after (bottom trace) 10 mg zolmitriptan in a single SCI participant in response to medial arch stimulation. Black bar denotes long-lasting reflex response (LLR: 500 ms after stimulation and beyond). **(B,C)** were taken from D'Amico et al. (2013a).

In addition to facilitating motoneuron PICs, serotonin and noradrenaline also regulate sensory transmission pre- and post-synaptically via the activation of Gq and Gi-coupled receptors. For example, activation of Gq-coupled 5-HT_2_ and NAα_1_ receptors on Ia and Ib *inhibitory* interneurons facilitates Ia reciprocal and Ia/Ib non-reciprocal inhibition (Jankowska et al., 2000; Hammar and Jankowska, 2003). Activation of Gi-coupled 5-HT_1_ and NAα_2_ receptors on the terminals of group I/II muscle, skin and high threshold pain afferents and on *excitatory* interneurons, such as propriospinal and group II interneurons, reduces transmission in these pathways (Jankowska et al., 1993, 1994; Rekling et al., 2000; Millan, 2002; Yoshimura and Furue, 2006; Jordan et al., 2008). Because there is a dramatic loss of serotonin and noradrenaline below a SCI, recent experiments have examined the effects of administering specific 5-HT_1_ and NAα_2_ receptor agonists to restore control over sensory afferent transmission to the motoneuron. As mentioned previously, a single-shock stimulation to a dorsal root produces a 1-s long EPSP as revealed during cell hyperpolarization (bottom trace, Figure 11Ai). This EPSP contains both a short-latency, short-duration component (short EPSP) and a longer-latency, longer-lasting component (long EPSP). At rest (−72 mV), the long EPSP produced by the dorsal root stimulation activates a CaPIC to produce a plateau potential and self-sustained firing or long-lasting reflex response (LLR, top trace Figure 11Ai). When the specific agonist to the 5-HT_1B/D_ receptor, zolmitriptan, was applied the long (but not short) EPSP was eliminated (bottom trace, Figure 11Aii) so that at rest, a plateau potential and LLR was not triggered (top trace, Figure 11Aii). Testing with multiple 5-HT_1_ receptor agonists/antagonists showed that sensory transmission is mainly produced by the specific activation of 5-HT_1B_ and 5-HT_1F_ receptors (Murray et al., 2011b). Application of an agonist to the NAα2 receptor, clonidine, also reduced the long sensory-evoked EPSP to prevent triggering of plateaus/LLRs (Rank et al., 2011). Both of these findings indicate that facilitation of Gi-coupled 5-HT_1_ and NAα_2_ receptors can reduce transmission of excitatory sensory pathways after SCI in rats.

Similar findings have been obtained in humans with motor complete SCI where oral intake of zolmitriptan (10 mg dose) suppresses the activation of (SLR, Figure 11B). The reduction of these SLRs resulted in reduced triggering of the LLR responses or spasms (Figure 11C), similar to the findings in the rat. The reduction in spasms was likely not mediated by a reduction in plateau potentials given that zolmitriptan does not affect the Na or CaPIC (Murray et al., 2011b). The mainly monosynaptic H-reflex was also reduced by zolmitriptan (D'Amico et al., 2013a). The same dose of zolmitriptan had similar effects on the H-reflex in uninjured control participants indicating that despite the decreased levels of serotonin below the motor-complete injury, the 5-HT_1_ receptors were not supersensitive. A tonic elevation in the transmission of sensory inputs after SCI suggests there is a low or absent level of basal 5-HT_1_ and NAα_2_ receptor activity below an injury. As such, it is unlikely that Gi-coupled 5-HT_1_ and NAα_2_ receptors display functional levels of constitutive receptor activity, unlike the Gq-coupled 5-HT_2_ and NAα_1_ receptors on motoneurons. Understanding why 5-HT_1_ and NAα2 receptors do not become constitutively active may help devise new strategies to control sensory transmission and spasticity after SCI.

Additional mechanisms producing enhanced sensory transmission to motoneurons after SCI are reviewed in Roy and Edgerton (2012) and include the suggestion by Kitzman (2005) that a decrease in dendritic number, without a subsequent increase in voltage-gated channels, may also increase the excitability of motoneurons after SCI. Moreover, elevations in motoneuron vesicular glutamate transporter 2 (2-IR), but not the 1-IR transporter on myelinated primary afferents, suggests that polysynaptic glutamatergic inputs from interneurons (but not from monosynaptic pathways) are elevated after SCI (Kitzman, 2006, 2007). In addition to neuronal changes, overexpression of glutamate receptor 1 (GluR1) on astrocytes has also been implicated in exacerbating sensory-evoked spasms in an ischemic model of rat SCI (Hefferan et al., 2007).

## Treatments for spasticity and recovery of motoneuron function

When involuntary muscle activity leads to impairments in function, difficulties in hygiene, skin breakdown and/or pain, treatment is warranted. As discussed above, spasticity is caused by adaptations to the muscle and spinal circuits as a result of a disruption in descending activation of the spinal cord. Most treatment strategies for spasticity target these secondary adaptations with limited success. Strategies that aim to fix the primary cause of spasticity by enhancing functional descending and/or peripheral activation of the spinal cord, in both motor complete and incomplete SCI, may prove to be more efficacious.

### Current pharmacological treatments

**Figure 12:**
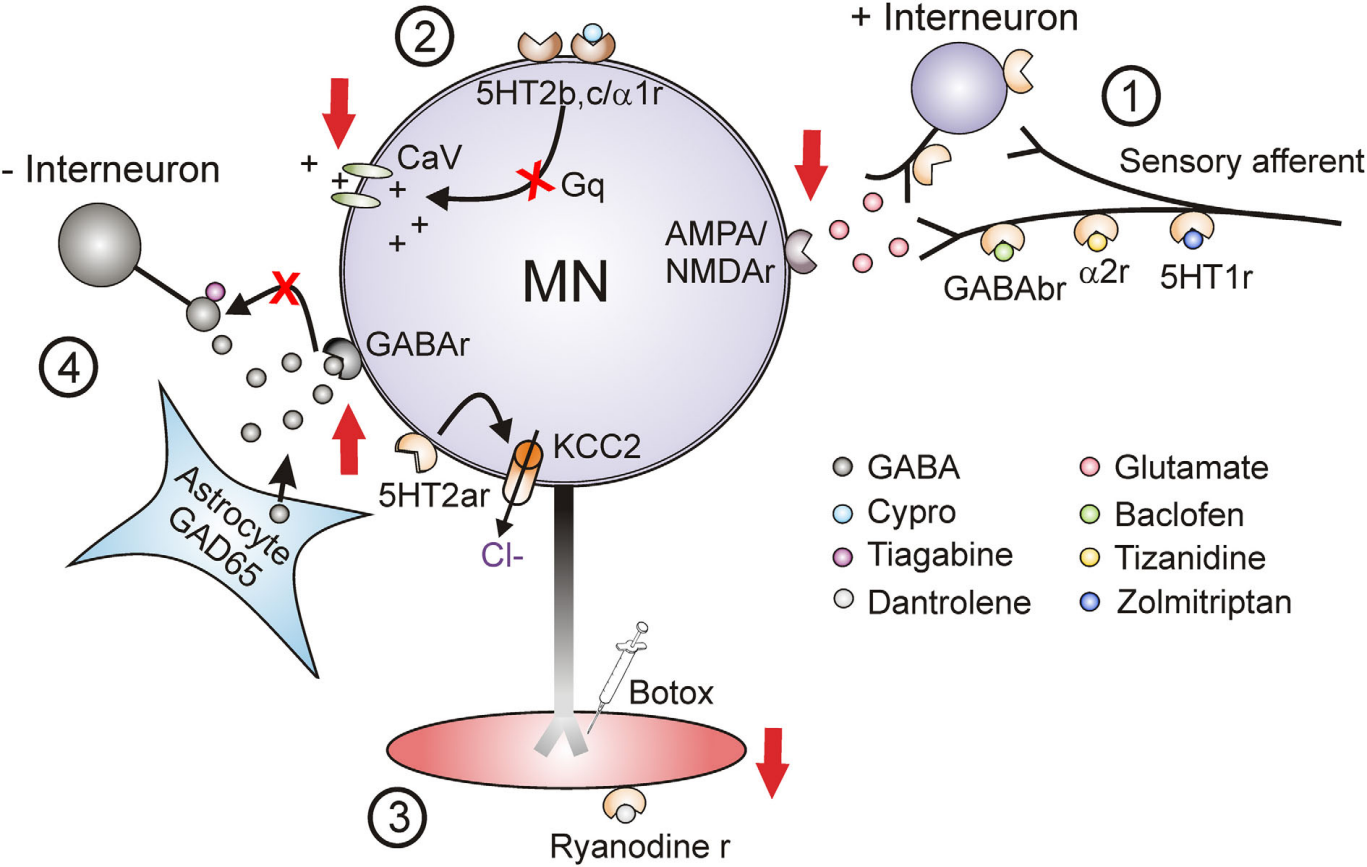
**Target sites for current and future antispastic treatments**. Pre-synaptic (1), motoneuron (2), muscle (3), and novel (4) sites for antispastic treatments. *Site 1:* GABA_B_, NAα_2_, and 5-HT_1_ receptors (r) located on pre-synaptic terminals or on interposed excitatory interneurons can be activated by baclofen, tizanidine, and zolmitriptan, respectively, to reduce excitatory glutamatergic release and activation of α-amino-3-hydroxy-5-methyl-4-isoxazolepropionic acid (AMPA) and *N*-methyl-D-aspartate (NMDA) receptors located on the motoneurons. *Site 2:* 5-HT_2_ and NAα_1_ receptors on the motoneuron with constitutive or ligand activation facilitate downstream voltage-gated calcium channels (CaV) mediating PICs via Gq protein-coupled pathways. Inverse agonists, such as cyproheptadine, switch the 5HT_2_ and NAα_1_ receptors to their inactive state, reducing activity in the Gq pathway and lessening activation of PICs and consequently muscle spasms. *Site 3:* Botulinum toxin (Botox®) injection directly into spastic muscles or activation of ryanodine receptors by dantrolene sodium (dantrolene) reduces spasticity by targeting the muscle site directly. *Site 4:* Spinal injection of the HIV1-CMV-GAD65 lentivirus increases GAD65 gene expression and GABA release from astrocytes. Systemic administration of tiagabine, a GABA reuptake inhibitor, increases amount of enhanced GABA in synapse to activate pre- and post-synaptic GABA receptors to reduce motoneuron excitability. Activation of 5-HT_2A_ receptors increases cell expression of KCC2 cotransporter to reduce intracellular concentration of Cl^−^ and increase endogenous motoneuron inhibition. Adapted from D'Amico et al. (2013a).

Current pharmacological treatments for spasticity aim to suppress spinal cord or muscle activity. Three main sites are targeted to reduce: (1) sensory transmission to motoneurons, (2) motoneuron excitability and (3) muscle contractions (Figure 12). Unfortunately when taken orally, these medications are not specific to the spinal cord or muscle and the majority produce some generalized depression of the CNS, resulting in altered mental states such as drowsiness and dizziness and in generalized weakness (Satkunam, 2003). For many of these agents long-term, high-dose use can also result in physiologic dependence (Simon and Yelnik, 2010) such that sudden withdrawal can result in serious consequences such as seizures (Grosset and Grosset, 2004). The most commonly prescribed agent for spasticity is baclofen, a GABA_B_ agonist which acts pre-synaptically (site 1, Figure 12) to reduce the amount of excitatory neurotransmitter released into synapses (Curtis et al., 1997; Li et al., 2004b). Although oral baclofen can reduce spasticity, it has detrimental effects on walking in patients with incomplete SCI, where other antispastics such as clonidine and cyproheptadine do not (Wainberg et al., 1986, 1990; Norman et al., 1998; Barbeau and Norman, 2003). Long-term use of oral baclofen also exacerbates the weakness and fatigability of motor units (Thomas et al., 2010). In cases of severe spasticity where patients are refractory to other treatments, baclofen is applied to the cerebrospinal fluid surrounding the spinal cord via an intrathecal pump (Lazorthes et al., 1990; Stempien and Tsai, 2000; Krach, 2001). Although intrathecal delivery reduces systemic side effects, it has a high rate (1%/month) of serious complications such as catheter obstruction which results in sudden withdrawal of baclofen and requires surgical intervention to repair (Draulans et al., 2013). Similar to baclofen, tizanidine (and clonidine) enhances pre-synaptic inhibition but via activation of NAα2 receptors on primary afferents and excitatory interneurons (site 1, Figure 12) (Davidoff, 1985; Krach, 2001). In one of the largest studies to date (78 SCI individuals), tizanidine reduced Ashworth scores by 37% in comparison to placebo (4%), with 81% of individuals reporting side effects (54% placebo) and no change in activities of daily living (Nance et al., 1994). Zolmitriptan also reduces sensory transmission by binding to 5-HT_1B_ receptors (site 1, Figure 12) (Murray et al., 2011b) but is not used as an oral anti-spastic given that regular use can induce headaches (Limmroth et al., 1999) and it is contraindicated in people with cardio-, cerebro-, or peripheral vascular disease (Peterlin and Rapoport, 2007). Diazepam, an allosteric modulator of the GABA_A_ receptor, both pre and post-synaptically (site 4) (Polc et al., 1974; Sieghart, 1994), and gabapentin, a GABA analog with unknown mechanism of action (Taylor, 1994; Sills, 2006) are also used as anti-spastics in SCI, but as with most of the under-powered studies in SCI, there is no quantitative evidence for a significant clinical effect (Taricco et al., 2006).

The only currently used antispastic that directly affects the excitability of the motoneuron (site 2, Figure 12) is cyproheptadine (Nance, 1994; Gracies et al., 1997); however, it lacks regulatory approval for this use. Cyproheptadine is as effective in reducing excessive muscle activity (17% reduction in Ashworth scores) compared to clonidine (22%) and baclofen (22%) (Nance, 1994), although this was only tested in 25 participants. Associated with the anti-histamine and anti-cholinergic properties of cyproheptadine were side effects such as CNS depression, dry mouth, appetite stimulation and thus, the potential for weight gain (Gracies et al., 1997). Despite these side effects, at low doses (12 mg/day) cyproheptadine decreased involuntary muscle spasms and clonus to improve residual walking function in participants with incomplete SCI (Wainberg et al., 1990). The development of more specific inverse agonists to 5-HT_2_/NAα_1_-receptors with fewer side effects than cyproheptadine may lead to better oral control of involuntary muscle spasms and clonus in SCI.

Reduction of spasticity by directly targeting the muscle (site 3, Figure 12) is achieved with botulinum toxin injected into specific muscles and with oral dantrolene sodium (Satkunam, 2003). Botulinum toxin, which blocks the release of acetylcholine at the neuromuscular junction, allows a more localized reduction in spasticity to injected muscles, but comes at the cost of further reducing muscle strength (Das and Park, 1989a,b; Simpson, 2004). Botulinum toxin produced stable improvements in upper-extremity function, hygiene and decreased pain in ~65% of individuals with SCI (Marciniak et al., 2008). However, the duration of effect is only 2–4 months, requiring repeated injections which may further increase the amount of non-contractile tissue within the target muscle (Fortuna et al., 2013). Dantrolene sodium is an oral antispastic that affects the excitation-contraction coupling in skeletal muscles by decreasing the release of calcium from the sarcoplasmic reticulum through blocking the ryanodine receptor (Britt, 1984; Krause et al., 2004; Lanner et al., 2010). In individuals with SCI, dantrolene sodium effectively reduced torque in response to passive stretch of the muscle (Herman et al., 1972; Glass, 1974; Glass and Hannah, 1974; Meyler et al., 1981), decreased reflex hyperexcitability and clonus and improvements in activities of daily life (Meyler et al., 1981). While CNS depression may occur with dantrolene sodium, it is less severe than other oral antispastics (Watanabe, 2009; Lapeyre et al., 2010). Despite the reduction in reflexes, dantrolene sodium is not commonly prescribed for SCI spasticity likely because it produces generalized muscle weakness (Adams and Hicks, 2005) and can result in hepatotoxicity (Gracies et al., 1997).

### New avenues for pharmacological and gene therapy treatments

A common disadvantage of all oral treatments is their action on supraspinal centers. Therefore, the ability to direct drug therapies specifically to the spinal cord is very desirable. Recently, a spinally targeted strategy was proposed by Marsala's group (site 4, Figure 12) where, in a rat model of ischemic SCI, the efficacy of a GABA uptake inhibitor tiagabine, applied systemically, was increased while localizing its effects to the lumbar spinal cord (Kakinohana et al., 2012). To do this, a lentivirus applied to the lumbar spinal cord locally upregulated GAD65 gene expression in astrocytes. Because of the enhanced production of GABA, the previously ineffective dose of tiagabine was now able to reduce hindlimb stretch reflexes without producing side effects such as CNS depression (Kakinohana et al., 2012). The use of spinally-targeted gene therapies opens up several different avenues for anti-spastic treatments with numerous potential targets, such as the constitutively-active isoforms of the 5-HT_2C_ receptor or the KCC2 transporter. Despite the promise of this strategy and its success in treating other disorders such as Parkinson's disease (Bjorklund et al., 2000; Kaplitt et al., 2007) and blindness (Dinculescu et al., 2005; Buch et al., 2008; Stieger et al., 2011), there are potential drawbacks to the use of gene therapies including small packaging capacity, the risk of insertional mutagenesis, immune responses against a vector and difficulty in supplying sufficient concentrations of the selected genes needed for human clinical trials (Monahan and Samulski, 2000). Thus, further research into this promising application is needed.

As discussed above, novel targets for reducing spasticity, such as the application of agonists to the 5-HT_1B/F_ receptors, like zolmitriptan, to reduce sensory transmission (site 1, Figure 12) and 5-HT_2A_ receptors to enhance KCC2 activity (site 4, Figure 12) are not suitable as oral antispastics due to their potentially harmful effects at supraspinal and cardiovascular centers. However, if these agonists were delivered intrathecally to the spinal cord, reductions in spasticity may be achieved over the long-term. It would be worthwhile examining in animal models of SCI if long-term use of intrathecal 5-HT_1/2A_ agonists is effective and if their symptoms of sudden withdrawal are less severe than with baclofen. Additionally, it would be worth testing the efficacy of intrathecal application of cyproheptadine which exerts its effects directly on the motoneuron by reducing 5-HT_2_ and NAα_1_ receptor activity. Because cyproheptadine reduces the excitability of the motoneuron directly, it may be used to treat spasticity for CNS disorders of varying etiology, such as amyolateral sclerosis, multiple sclerosis, stroke, traumatic brain injury and cerebral palsy. Better technologies for intrathecal or spinal drug delivery will open possibilities for the application of these newly discovered antispastics. However, as discussed above, any agents that suppress spinal cord excitability may further promote the compensatory changes within the spinal cord that increases its excitability, such as increases in constitutive monoamine receptor activity and sensory transmission and decreases in KCC2 activity. In fact, the development of tolerance to antispastic drugs may, in part, be produced by increasing these compensatory mechanisms by pharmacological suppression of the spinal cord and muscle. Thus, strategies which enhance functional synaptic activation of the spinal cord, such as the physical treatments described below, may be the best solution to curb hyperexcitabilty of the spinal cord after injury.

### Physical treatments

Physical treatments can successfully reduce spasticity and they avoid the negative side effects associated with pharmacological treatments. The first line of prevention and treatment of spasticity by physical treatments targets the maintenance of joint range of motion through strategies such as stretching (Bovend'Eerdt et al., 2008), range of motion exercises (Harvey et al., 2009a), static weight bearing (Odeen and Knutsson, 1981), and passive cycling (Rayegani et al., 2011). All of these techniques have produced significant reductions in measures of spasticity such as the Ashworth score and the pendulum test. However, these techniques have produced inconsistent results on range of motion and there is sparse evidence regarding improvements in motor function (Harvey et al., 2009b), though reductions in co-contraction and improved gait from stretching have been reported (Bovend'Eerdt et al., 2008). Physical treatments produced a reduction in atrophy-related changes in muscle architecture (Willoughby et al., 2000), reduced passive stiffness (Guissard and Duchateau, 2004), reduced Ia transmission as assessed by Hmax/Mmax ratios (Guissard and Duchateau, 2004; Rayegani et al., 2011) and normalization of motoneuronal excitability as assessed by F-wave/M-wave ratios (Rayegani et al., 2011) to potentially contribute to the observed reductions in spasticity.

In participants with incomplete SCI who can perform voluntary contractions, therapies initiated to improve motor function can also reduce spasticity. For example, body-weight supported treadmill training consistently reduces ankle clonus across various studies, in addition to reducing flexor and extensor spasms and co-contraction and increasing PAD_2_ (Gorassini et al., 2009; Adams and Hicks, 2011; Manella and Field-Fote, 2013; Knikou and Mummidisetty, 2014). These measures of spasticity are also associated with improvements in motor control such as increases in voluntary motor strength, walking speed, and self-reported mobility. In a recent pilot study, resistance training at maximal intensity also improved walking and balance with a trend to decrease the modified Ashworth score (Jayaraman et al., 2013). Other forms of therapy involving voluntary contractions include neural facilitation, hippotherapy and hydrotherapy, and all have resulted in reduced measures of spasticity (Hsieh et al., 2012). For example, 10 weeks of hydrotherapy (3 × 's per week), in addition to passive range of motion exercises, significantly decreased the Ashworth and Penn spasm frequency scores and resulted in a significant decrease in the dosage of oral baclofen taken compared to passive exercises alone (Kesiktas et al., 2004). Increases in the strength of spared corticospinal pathways have also been produced from physical treatments that incorporate voluntary contractions, such as treadmill training (Thomas and Gorassini, 2005). Thus, reductions in spasticity from these active physical treatments that provide functional, patterned synaptic activation of the spinal cord may result from enhanced activation of spinal inhibitory circuitry by strengthened descending motor pathways (Zewdie et al., 2011) and/or from decreases in sensory transmission by enhanced activation of the dorsal horn by descending monoaminergic pathways (Murray et al., 2011b; Rank et al., 2011). In addition, the increased activation of the spinal cord by synaptic inputs during active treatments may increase spinal BDNF to facilitate KCC2 activity and subsequently, intrinsic motoneuron inhibition (Gomez-Pinilla et al., 2007; Boulenguez et al., 2010).

Electrical stimulation of the spinal cord, peripheral nerves and muscles has also been used to treat spasticity and/or motor impairment. A single session of stimulation that results in muscle contractions consistently demonstrates acute reductions in spasticity, which is thought to be mediated by muscle fatigue induced from electrically-elicited contractions (Marsolais and Kobetic, 1988). For example, stimulation of the triceps surae generating isometric contractions decreased the modified Ashworth score for the plantarflexors (Van Der Salm et al., 2006). Similarly, functional electrical stimulation (FES)-assisted cycling also decreased the modified Ashworth score and pendulum test in people with complete SCI (Krause et al., 2008). It has been theorized that long-term stimulation which increases muscle hypertrophy (Skold et al., 2002) and strength (Granat et al., 1993) could be associated with increases in spasm strength. However, research has failed to consistently confirm that long-term stimulation protocols adversely impact spasticity. In contrast, an 8 week program of stimulation of the quadriceps and hamstrings that produced subtetanic isometric contractions reduced spasms as measured by SCATs in people with motor complete SCI (Carty et al., 2013). Similarly people with incomplete SCI who used FES to augment gait training for at least 16 months had decreased intrinsic and reflex stiffness (Mirbagheri et al., 2002). Moreover, 15 days of tibial nerve stimulation that did not result in perceptible contractions reduced Ashworth scores, spasm frequency, tendon and H-reflex to a similar extent as oral baclofen (Aydin et al., 2005). Although most studies show improvements in spasticity with long-term electrical stimulation, some individuals did rank their spasticity as worse following 6 months of FES cycling, even though there were no changes in their modified Ashworth Scores (Skold et al., 2002). More invasive modes of stimulation, such as spinal cord stimulation with epidural electrodes, improved resistance to passive stretch and reduced the use of antispastic medications (Pinter et al., 2000). However, some negative side effects with a previous epidural stimulator included loss of effectiveness and stimulator failure over ~10 year period (Midha and Schmitt, 1998) and suggest, like intrathecal drug delivery, some technical issues need improvement before more invasive methods of treating spasticity become mainstream.

### Combined excitatory pharmacological and physical treatments

An alternative method to reduce spasticity may be to first *increase* the functional activation of the spinal cord by residual descending and intact peripheral synaptic inputs. This would then increase control over excitatory circuits and enhance the activation of intrinsic inhibitory mechanisms (KCC2) to curb involuntary, intrinsic (PIC) activation of motoneurons. Recently, combined excitatory pharmacological and physical treatments have been used in both animals and humans to increase spinal cord excitability and promote the recovery of walking function. In participants with *incomplete* SCI, treadmill training was combined with the oral administration of escitalopram, a selective serotonin reuptake blocker, to increase the amount of serotonin released in the spinal cord (Hornby et al., 2009). Elevation in serotonin levels would potentially increase the excitability of motoneurons via 5-HT_2B/C_ receptors (Murray et al., 2011a), increase the excitability of locomotor interneurons via 5-HT_1A_, 5-HT7, and 5-HT_2A/C_ receptors (Courtine et al., 2009; Jordan and Slawinska, 2011; Slawinska et al., 2013) and decrease transmission of sensory inputs via 5-HT_1B/F_ receptors (Murray et al., 2011b). When combined with 8 weeks of treadmill training, escitalopram produced a greater improvement in walking function (maximum speed) when compared to participants receiving treadmill training alone (Hornby et al., 2009). Likewise, a combination of spinal cord stimulation and intrathecal application of 5-HT_1A_, 5-HT_7_, and 5-HT_2A_ receptor agonists improved volitional control of walking in a rat model of incomplete SCI (Van Den Brand et al., 2012). In the human study, although walking performance improved with increases in 5-HT receptor activation, measures of spasticity also increased (Thompson et al., 2011; Thompson and Hornby, 2013), likely due to the facilitation of 5-HT_2B/C_ receptors. It remains to be seen if, over time, motor training can better shape the enhanced activation of the spinal cord by descending and peripheral inputs to improve control of spasticity when excitability of the spinal cord is increased pharmacologically. Perhaps application of 5-HT receptor agonists that specifically target locomotor-related interneurons (e.g., 5-HT_1A_, 5-HT_7_, and 5-HT_2A_) over motoneurons (5-HT_2B/C_) may produce greater improvements in walking function with fewer increases in spastic motor behaviors.

## Conclusions

Following the reduction of synaptic excitation by descending and movement-related sensory inputs, the spinal cord below the injury adapts by increasing the intrinsic excitability of motoneurons via increases in constitutive monoamine receptors and downregulation of KCC2, resulting in uncontrolled activation of muscles below the injury. Strategies to help improve controlled and functional activation of motoneurons via synaptic inputs may help to strengthen the purposeful activation of muscles and reduce the involuntary activation of motoneurons by suppressing some of these maladaptive changes. For example, enhanced descending and/or sensory activation of motoneurons during facilitated motor tasks, such as treadmill or exoskeleton walking, FES-assisted cycling or rowing, which can be applied to participants with motor complete or incomplete injuries, may produce enough pattered activation of motoneurons to help curb, in an activity dependent manner, increases in constitutive monoamine receptor activity and/or promote BDNF-facilitation of KCC2 activity (Boulenguez et al., 2010). Thus, rather than suppressing the involuntary activation of motoneurons and muscles with suppressive pharmacology, perhaps we should be exciting the spinal cord with functional, patterned activity that occurs, or mimics, inputs produced during natural movement to keep intrinsic, inhibitory mechanisms viable. Despite the numerous and effective physical strategies to improve motor function and treat spasticity, individuals with SCI are typically prescribed pharmacological interventions that reduce spinal cord excitability as the first line of treatment (Rekand et al., 2012). Further studies comparing the effectiveness of physical, vs. suppressive pharmacological, treatments over the long-term are needed to determine the best approach for treating and managing spasticity after SCI. The best approach to control spasticity and improve residual motor function may be to increase the activation of the spinal cord with functional, patterned inputs akin to what happens during natural movements such as walking and reaching. This may improve both descending and peripheral control over excitatory spinal circuits and enhance intrinsic inhibition in the spinal cord to reduce spasticity.

### Conflict of interest statement

The authors declare that the research was conducted in the absence of any commercial or financial relationships that could be construed as a potential conflict of interest.
