# Corrigendum: Recovery of neuronal and network excitability after spinal cord injury and implications for spasticity

**DOI:** 10.3389/fnint.2014.00049

**Published:** 2014-06-16

**Authors:** Jessica M. D'Amico, Elizabeth G. Condliffe, Karen J. B. Martins, David J. Bennett, Monica A. Gorassini

**Affiliations:** ^1^Centre for Neuroscience, University of AlbertaEdmonton, AB, Canada; ^2^Faculty of Medicine and Dentistry, University of AlbertaEdmonton, AB, Canada; ^3^Department of Biomedical Engineering, University of AlbertaEdmonton, AB, Canada; ^4^Division of Physical Medicine and Rehabilitation, University of AlbertaEdmonton, AB, Canada; ^5^Faculty of Physical Education and Recreation, University of AlbertaEdmonton, AB, Canada; ^6^Faculty of Rehabilitation Medicine, University of AlbertaEdmonton, AB, Canada

**Keywords:** spasticity, motoneurons, spinal cord injury, rehabilitation, reflex

In Figure 10 of D'Amico et al. (2014) the KCC2 co-transporter was incorrectly drawn as a co-exchanger. This has been corrected in this version of the figure to show that both chloride and potassium are pumped out of the motoneuron.

**Figure 10:**
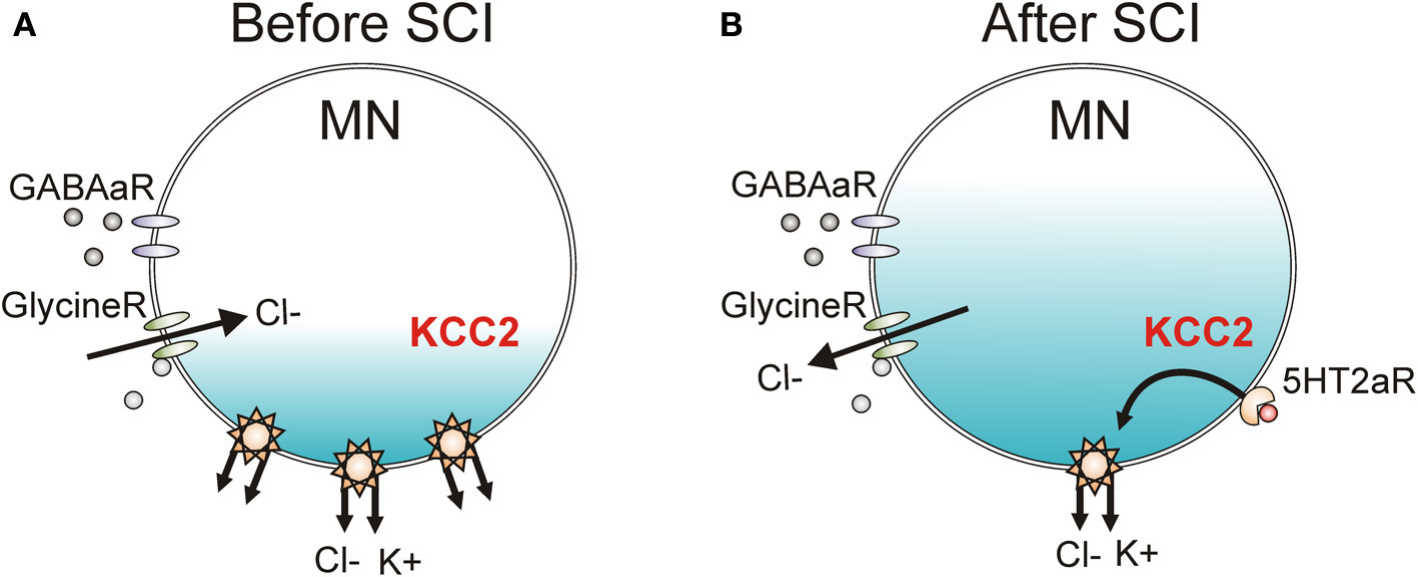
**KCC2 cotransporter and chloride equilibrium before and after SCI. (A)** A potassium chloride cotransporter (KCC2) transports both chloride (Cl^−^) and potassium (K^+^) out of the motoneuron (MN) to maintain Cl^−^ equilibrium potential below resting membrane potential, allowing Cl^−^ influx and MN hyperpolarization during activation of GABA and Glycine receptors (R). **(B)** Downregulation of KCC2 expression in motoneurons after SCI increases intracellular Cl^−^ concentration, depolarizing Cl^−^ equilibrium potential to above rest. This produces efflux of Cl^−^ and depolarization of MN during activation of GABA and Glycine receptors. Activation of 5-HT2A receptors increases cell membrane expression of KCC2 after SCI to restore endogenous inhibition.

## Conflict of interest statement

The author declares that the research was conducted in the absence of any commercial or financial relationships that could be construed as a potential conflict of interest.

